# Morphological Engineering of Sensing Materials for Flexible Pressure Sensors and Artificial Intelligence Applications

**DOI:** 10.1007/s40820-022-00874-w

**Published:** 2022-07-05

**Authors:** Zhengya Shi, Lingxian Meng, Xinlei Shi, Hongpeng Li, Juzhong Zhang, Qingqing Sun, Xuying Liu, Jinzhou Chen, Shuiren Liu

**Affiliations:** 1 School of Materials Science and Engineering, Henan Key Laboratory of Advanced Nylon Materials and Application, Henan Innovation Center for Functional Polymer Membrane Materials, Zhengzhou University, Zhengzhou, 450001 People’s Republic of China; 2 Wenzhou Institute, University of Chinese Academy of Sciences, Wenzhou, 352001 People’s Republic of China; 3 School of Mechanical Engineering, Yangzhou University, Yangzhou, 225127 People’s Republic of China

**Keywords:** Flexible pressure sensor, Morphological engineering, Sensing performance, Manufacturing technique, Artificial intelligence

## Abstract

- Various morphological structures in pressure sensors with the resulting advanced sensing properties are reviewed comprehensively.
- Relevant manufacturing techniques and intelligent applications of pressure sensors are summarized in a complete and interesting way.
- Future challenges and perspectives of flexible pressure sensors are critically discussed.

## Abstract (highlights)

As an indispensable branch of wearable electronics, flexible pressure sensors are gaining tremendous attention due to their extensive applications in health monitoring, human –machine interaction, artificial intelligence, the internet of things, and other fields. In recent years, highly flexible and wearable pressure sensors have been developed using various materials/structures and transduction mechanisms. Morphological engineering of sensing materials at the nanometer and micrometer scales is crucial to obtaining superior sensor performance. This review focuses on the rapid development of morphological engineering technologies for flexible pressure sensors. We discuss different architectures and morphological designs of sensing materials to achieve high performance, including high sensitivity, broad working range, stable sensing, low hysteresis, high transparency, and directional or selective sensing. Additionally, the general fabrication techniques are summarized, including self-assembly, patterning, and auxiliary synthesis methods. Furthermore, we present the emerging applications of high-performing microengineered pressure sensors in healthcare, smart homes, digital sports, security monitoring, and machine learning-enabled computational sensing platform. Finally, the potential challenges and prospects for the future developments of pressure sensors are discussed comprehensively.

## Graphical abstract

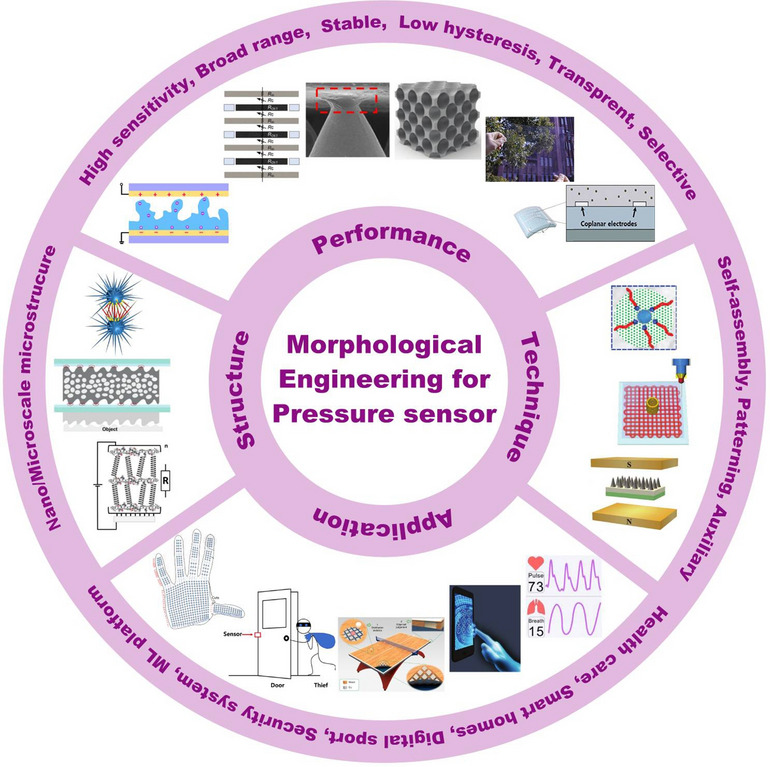

## Introduction

Driven by market speculation, trends among youth, and massive funding, wearable technology has become a topic of interest in academia and industry because of broad applications in growing disruptive fields, such as wearable medical devices [1–7], artificial intelligence (AI) [8–16], and the Internet of Things (IoT) [17–24]. Flexible pressure sensors that convert pressure into electrical output are an essential part of flexible electronics. Due to rapidly advancing techniques in electrical sensing, material sciences, system engineering, and signal processing, flexible pressure sensors have been investigated in multiple disciplines because of their unique advantageous properties, such as outstanding flexibility, low cost, and compatibility with large-area processing techniques [25–29]. For example, flexible pressure sensors can be attached to the human body for real-time monitoring of different physiological parameters like blood pressure [2, 30], blood flow [1, 30], pulse beat [2, 6, 31, 32], heartbeat [32], respiration [31, 32], tremor [33–35], and body movement [36–39]. These parameters are of vital importance in biomedical research, disease diagnosis, and timely treatment. Interest in integrated networks of sensors has been motivated by promising applications in intelligent robotics [40–42], human–machine interactions (HMI) [34, 35, 43, 44], biomimetic prostheses [4, 45–47], smart homes [48–50], digitizing sports [20, 21, 51, 52], wireless monitoring in security [17, 23, 44, 53], and machine learning (ML)-enabled computational sensing platforms [54–60], promoting the advancement of AI systems. Substantial achievements have been made for pressure sensors, including but not limited to piezoresistive [37, 61–63], piezocapacitive [64–66], transistor [48, 67, 68], piezoelectric [25, 49, 69], and triboelectric sensors [31, 70–73].

Some criteria have been employed to evaluate the performance of pressure sensors, including sensitivity [74–78], working range [35, 50, 79], stability [80–83], hysteresis [84], response and recovery time [85]. In practical applications, it is desirable to utilize pressure sensors with high sensitivity to distinguish subtle pressure changes, a wide working range for broad detection applicability, and a fast response and low hysteresis to monitor high-frequency pressure and obtain accurate measurements. Therefore, various sensing materials with well-suited morphological microstructures have been investigated to achieve large conductive contact changes [61, 86, 87], effective stress concentration [74], and signal conduction [83] in the sensing layer [25, 48, 62, 75, 88] and electrode [66, 89–93]. These microstructures commonly occur at the nanometer and micrometer scale, including variable energy bands [94], and tunable layer spacing [95–97], cracks [98–101], microroughness [93, 102, 103], porous hierarchical structure [32, 34, 37, 61, 104–106], and multiscale hierarchical structure [25, 63, 74, 82, 107–110]. The microstructure is fabricated using (attractive [111–113] or repulsive [26, 114]) self-assembly, (lithography [115], patterning [116], polymerization [117]) patterning, and (mechanical force [66], electronic field [118], magnetic field [119], gas-bubbling processing [64, 120], template confinement [121]) auxiliary manufacturing technologies.

**Fig. 1:**
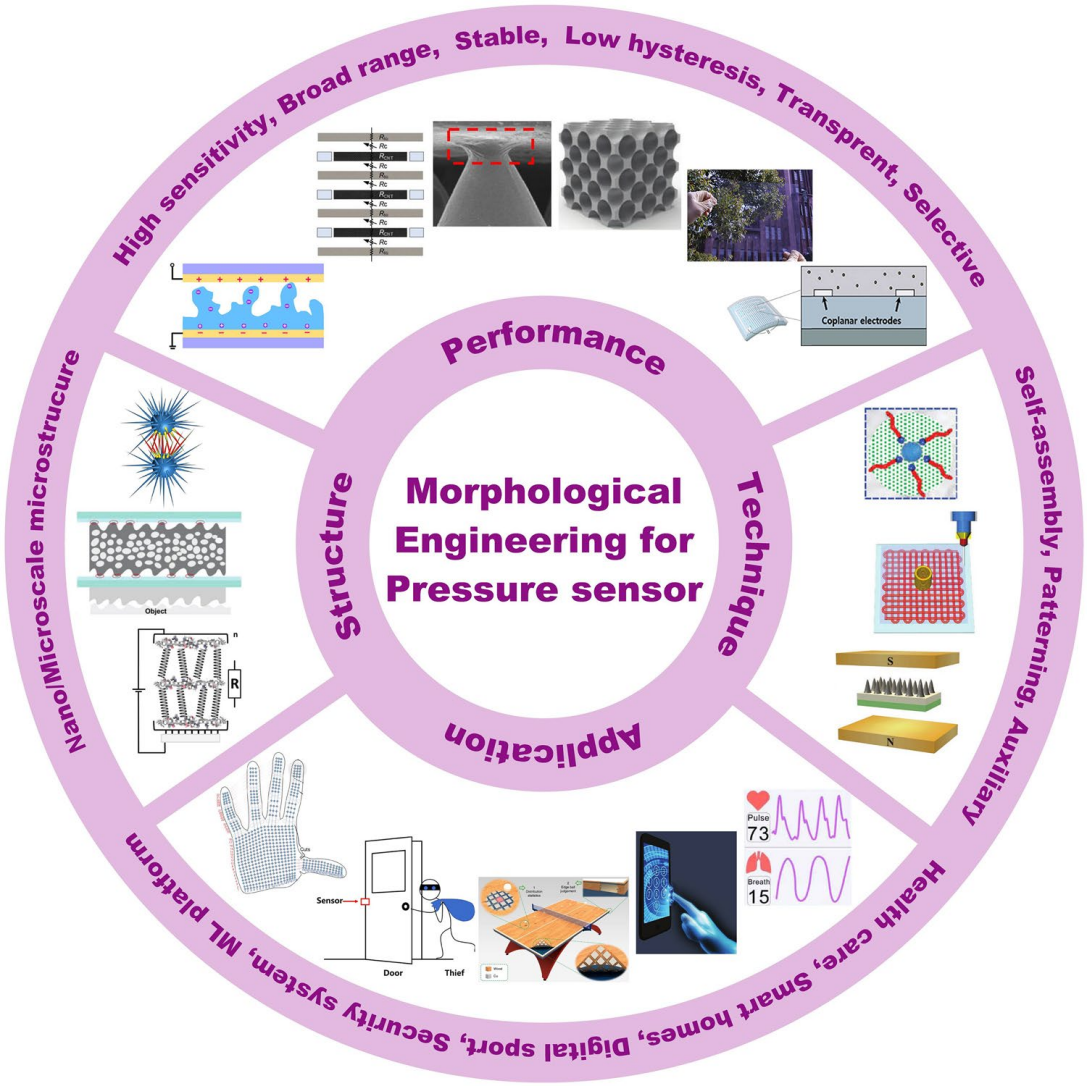
Summarization of structure, performance, technique and application for flexible micromorphological pressure sensor. Sensing materials with variable nanometer-scale microstructure [96]. Copyright (2018) Wiley–VCH, micrometer-scale microstructures [74, 108]. Copyright (2018) The Authors and (2019) Wiley–VCH. Performance: high sensitivity [75] (Copyright (2020) The Authors), broad working range [50] (Copyright (2019) Wiley–VCH), stable sensing [81] (Copyright (2022) The Authors), low hysteresis [84] (Copyright (2019) Wiley–VCH), high transparency [226] (Copyright (2016) Springer Nature), directional-selective sensing [227] (Copyright (2018) Wiley–VCH). Technique: self-assembly [26] (Copyright (2014) Wiley–VCH), patterning [116] (Copyright (2017) Wiley–VCH), auxiliary synthesis [119] (Copyright (2020) Wiley–VCH). Application: wearable electronics in health care [31] (Copyright (2020) The Authors), intelligent device in smart homes [72] (Copyright (2020) Wiley–VCH), digital sports [21] (Copyright (2019) The Authors), wireless monitoring in security system [53] (Copyright (2020) American Chemical Society). ML-enabled intelligent sensing platform [57] (Copyright (2019) Springer Nature)

Several reviews have summarized the progress in flexible pressure sensors, focusing on flexible electronics [68, 122] and emerging materials (e.g., graphene-based [123, 124], biological materials [125]). Additionally, the structure design of pressure sensors has been addressed in some of them, such as three-dimensional (3D) monolithic conductive sponge [126], microstructures geometrical design [38, 125, 127, 128], microengineering of sensing layer with geometric features ≈ 1–1000 µm in size [129]. However, there is still an urgent need to comprehensively summarize the progress of morphological engineering of sensing material in pressure sensors as they may provide additional insights on compatibility and sensing performance such as sensitivity, limit of detection, dynamic range, stability, and response and relaxation times. Therefore, this review provides a holistic view of recent developments in the morphological engineering of flexible pressure sensors. A brief introduction of the transduction mechanisms is described in Sect. 2. Commonly used micromorphological structures are reviewed in Sects. 3 and 4, with an emphasis on the design of high-performance sensing capacities. Section 5 summarizes general preparation technologies for these interesting microstructures. In Sect. 6, current state-of-the-art flexible pressure sensors used in wearable electronics, smart homes, and data monitoring and security applications are presented. The conclusions and future prospects are provided in the final section. The structure of this review and the relationships between the parts are shown in Fig. 1. We hope that this review can provide inspiration for the design of advanced pressure sensors.

## Sensing Mechanisms

**Fig. 2:**
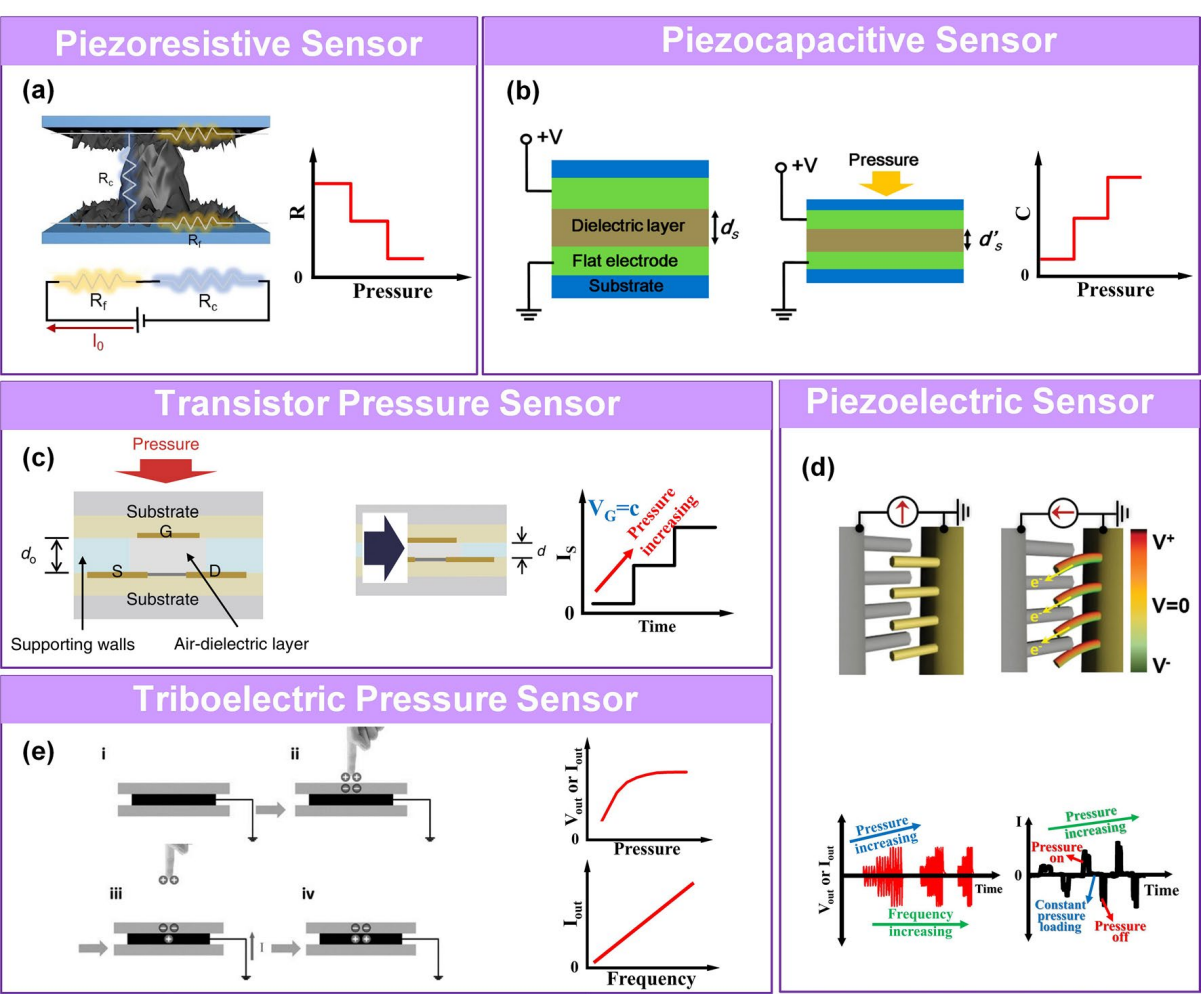
Schematic diagrams and sensing characteristics for **a** piezoresistive [63]. (Copyright (2020) American Chemical Society), **b** piezocapacitive [66] (Copyright (2017) American Chemical Society), **c** transistor [68] (Copyright (2017) The Authors), **d** piezoelectric [25] (Copyright (2015) Wiley–VCH), and **e** triboelectric pressure sensor [70] (Copyright (2017) Wiley–VCH)

Pressure sensors can be classified as piezoresistive [63], piezocapacitive [66], transistor [68], piezoelectric [25], and triboelectric [70] types (Fig. 2). Each of these sensing mechanisms has unique characteristics due to the active materials and device structure. A brief review of the types of sensing mechanisms is presented in this section.

### Piezoresistive Sensor

Piezoresistive sensors have been extensively investigated owing to their simple device structure, relatively broad working range, low operating voltage, low energy consumption, ease of fabrication, signal collection, and readout [130]. The principle of piezoresistive sensors is based on transducing the external pressure stimulus applied to the device into a recordable resistance value [63], consisting of the contact resistance at the interface between the electrode and the sensing material, the interior resistance of the sensing material and the electrode. The resistance of the material can be expressed by the following equation:

1 $$R = \frac{\rho L}{A}$$

where *R* is the resistance of the material, *ρ* is the resistivity, *L* is the length, and *A* is the cross-sectional area. Commonly used sensing materials for piezoresistive sensors are conductive carbon material (e.g., carbon nanotubes (CNTs) [50, 131], graphene [132], MXene [62], carbon black (CB) [133], carbonized silk [134] carbonized wood [61], and carbonized crepe paper [110]), conductive polymer (poly(3,4-ethylenedioxythiophene):poly(styrenesulfonate) (PEDOT:PSS) [37], polypyrrole (PPy) [82], polyaniline (PANI) [111]), metal nanowire (NW) [135–138], nanoparticle (NP) [139] and film [140] (e.g., AuNW [136, 138], Ag NW [141], Cu NW [142], Pd NPs [139], Pt film [140]), metal-oxide (Fe_2_O_3_ [78], ZnO [74], SnO_2_ [143], In_2_O_3_ [143], NiO [143]), liquid metal [144, 145], and metal–organic frameworks (MOFs) [146]. The internal microstructure of the sensing material [102] and the electrode [89, 147] includes cracks [98, 99], micro-rough structures [93, 102, 103, 148], porous hierarchical structures [104, 106, 149], and multiscale hierarchical structures [74, 82, 107–110]. These structures improve the sensing performance by increasing the space of contact change and delaying contact saturation. Each part of the active material is considered a small resistance in the circuit model. The total resistance of the active material is regarded as being parallel in most cases, and the resistance of the active layer and the electrode part are in series [150, 151]. The sensitivity of piezoresistive sensors is defined as the ratio of the relative rate of change of the resistance or current to the applied pressure [79].

### Piezocapacitive Sensor

In general, piezocapacitive pressure sensors consist of an insulating medium (dielectric) sandwiched by two parallel conducting plates. Piezocapacitive pressure sensors operate by measuring the capacitance change of the parallel plate capacitor under pressure, which is expressed by the equation capacitance:

2 $$C = \frac{{\varepsilon_{r} \varepsilon_{0} A}}{d}$$

where *A* is the overlapping area of the two plates or the effective area of the capacitor, *ε*_*0*_ is the vacuum permittivity, *ε*_*r*_ is the relative permittivity of the dielectric material between the plates, and d is the separation between the plates. The capacitance depends on the plate spacing, contact surface, and effective dielectric constant affected by deformation under pressure [66]. A variety of conductive materials and composites have been developed as electrode materials for piezocapacitive sensors during the last decade, including Au film [75, 152, 153], Ag NWs [66, 154, 155], Ag NPs [65], CNTs [156], graphene [64], MXene nanosheets [157], PEDOT:PSS [158], and ionically conductive hydrogel [159]. Various low-modulus dielectric materials, such as polyvinylidene fluoride (PVDF) [66], Ecoflex [118, 154], polydimethylsiloxane (PDMS) [65, 160], polyurethane (PU) [161], and ionic liquid or gels (1-ethyl-3-methylimidazolium trifluoromethanesulfonate ([EMIM][OTF]) [161], PVA/H_3_PO_4_ [75]), have been investigated as dielectric media. The main advantages of capacitive sensors are a stable sensing performance, good dynamic response, and low power consumption. In addition, some piezocapacitive pressure sensors can work in a non-contact mode due to the pseudo-capacitance effect [89]. However, due to the viscoelasticity of polymer dielectric layers, these sensors typically have a relatively slow response speed.

### Transistor Sensor

Pressure-induced effects regulating the flow of carriers between the source and drain flow control is the mechanism of transistorized pressure sensors. It can be a pressure-induced change in dielectric layer capacitance [68] (with the commonly used materials of polystyrene-block-poly (2-vinylpyridine) (PS-*b*-P2VP) [162], PDMS/rubrene [163], PDMS/PiliTSi [164]) or a change in gate voltage due to piezoelectric voltage or frictional voltage generated in a hybrid pressure sensor of piezoelectric transistor [67] or tribotronic transistor [165] with instead gate electrode materials (such as piezoelectric material of poly(vinylidene fluoride/trifluoroethylene) P(VDF-TrFE) [166] and ZnO [67]; triboelectric material of polyimide (PI) [165] and polytetrafluoroethylene (PTFE) [167]). The sensitivity is expressed as the ratio of relative change of current between source and drain electrodes (ΔI_DS_/I_0_) to the change of applied pressure (δP). Transistor pressure sensors typically consist of high-resolution arrays, which can be used for pressure mapping distribution [165, 168]. Additionally, due to the amplification effect [79] of the transistor, a very small pressure load can induce a large change in the electrical signal, resulting in high sensitivity and low power consumption.

### Piezoelectric Sensor

A piezoelectric pressure sensor operates on the principle of piezoelectricity and responds to external pressure by generating instantaneous electrical signals. Piezoelectricity is caused by the oriented and permanent dipoles in the piezoelectric material. Generally, when external pressure is applied to the device, the deformation of the oriented non-centrosymmetric crystal structures leads to the spatial separation of the positive and negative charges, resulting in charges on the cathode and anode. Commonly used piezoelectric sensing materials include piezoelectric crystals (lead zirconium titanate (PZT) [169, 170], gallium nitride (GaN) [171], BaTiO_3_ (BTO) [172], zinc oxide (ZnO) [25], aluminum nitride (AlN) [173]), piezoelectric polymers (PVDF [174–176], cellular polypropylene [177], fluorinated ethylene propylene (FEP) [2, 32], cyclic olefin copolymer (COC) [178]), bioderived piezoelectric materials [125] (onion skin [179], spider silk [180], eggshell [181]), piezoelectric peptide and metabolite materials [182] (diphenylalanine (FF) [183], β glycine [184], cyclo-glycine-tryptophan (cyclo-GW) [185]). Piezoelectric sensors are widely used to detect pressure and high-frequency vibration dynamically due to their high sensitivity and transient sensing ability. Their operation is similar to the rapid and dynamic contact perception ability of Meissner corpuscles and Pacinian corpuscles in the human skin [25, 186]. Since the output voltage of piezoelectric sensors is pulsed, they are not suitable for the measurement of static pressure.

### Triboelectric Sensor

Triboelectric pressure sensors have been extensively studied in recent years since the discovery of the principle by Wang and co-workers in 2012 [187]. They operate based on the coupling effect of electrostatic induction and contact electrification to induce polarization and produce voltage when pressure is applied. The accumulated electrical potential difference between the two counterparts causes the flow of electrons and generates a displacement current*.* The output signal of a triboelectric pressure sensor is related to the contact force, speed, and area, as well as the material properties. Triboelectric nanogenerators (TENGs) are considered promising candidates for pressure sensors due to their high-power output at low-frequency mechanical energy (< 5 Hz) [188], low cost, and simple preparation process. They operate in in four modes: 1) vertical contact separation, 2) single-electrode mode, 3) lateral sliding mode, and 4) freestanding triboelectric layer mode [189]. The first two modes are generally employed for pressure sensors. TENGs can monitor static and dynamic pressures by changing the measurement strategy. Open-circuit voltage with transferred charge density is required for static pressure detection. Piezoelectric sensors and triboelectric sensors are self-powered sensors whose sensitivity is defined as a change in the output current or output voltage under pressure [31]. Since the triboelectric effect is common in various materials, there are no distinct limitations on the materials used for piezoelectric sensing. Numerous advanced materials with high output performance have been utilized for triboelectric pressure sensors, including wood [21], paper [23], poly-ε-caprolactone [190], PTFE [191], PDMS [192], Kapton [73], nylon [193], cotton [73], FEP [44], acrylic [73], PU [70], polyvinyl chloride (PVC) [194], polyethylene (PE) [195]. These materials overall trend of friction polarity of positive triboelectric material series of PU > hair > nylon > glass > paper > pine wood > cotton > nitrile rubber, negative triboelectric material series of acrylic < PI < silicones < PE < PVC < PTFE [73]. The polarity of the ability of materials substantially affects the output performance.

The sensing materials, the advantages and limitations of the different types of pressure sensors are summarized in Table 1.

**Table 1:** Summary of sensing materials, uniqueness and limitations for pressure sensors

Mechanism	Sensing materials	Advantages and limitations
Piezoresistive	(CNTs [131], graphene [132], MXene [62], CB [133], carbonized silk [134] and carbonized wood [61]), conductive polymer (PEDOT:PSS [37], PPy [82], PANI [111]), metal nanowire [135–138], nanoparticle [139] and film [140] (Au NW [136, 138], Ag NW [141], Cu NW [142], Pd NP [139], Pt film [140]), metal-oxide (ZnO [74], Fe_2_O_3_ [78], SnO_2_ [143], In_2_O_3_ [143], NiO [143]), liquid metal [144, 145], and MOFs [146]	Simple device structure High sensitivity in low pressure area High power consumption Easy to drift
Capacitive	PVA/H_3_PO_4_ [75], SBS-Ag NP/PDMS [65], PVDF/PDMS-AgNW [66], PDMS/graphene [64], PDMS/Au film [153], SWNT-PDMS [156], Ecoflex/Ag NW [154], MXene/PVDF-TrFE [158], PDMS/Ag NW [160], PU-PVA/[EMIM][OTF] [161]	Can non-contact testing [89] Vulnerable to affected by surroundings
Transistor	(PS-*b*-P2VP) [162], PDMS/rubrene [163], PDMS/PiliTSi [164], MoS_2_/Al_2_O_3_ [279], PDMS/air [68], SiO_2_/Ag NF-Ag NW [48], Al_2_O_3_/HfO_2_ [168]	High resolution with array Low power consumption Complex installation layout
Piezoelectric	Piezoelectric crystals (PZT [169, 170], GaN [171], BTO [172], ZnO [25], AlN [173]), piezoelectric polymers (PVDF [174–176], cellular polypropylene [177], FEP [2, 32], COC [178]), bioderived piezoelectric materials [125] (onion skin [179], spider silk [180], eggshell [181]), piezoelectric peptide and metabolite materials [182]	Can percept high-frequency pressure and force direction [186] Not suitable for static pressure sensing [186]
Triboelectric	No special limitation for active materials, including positive triboelectric material (such as PU [70], nylon [193], paper [23], wood [21], cotton) and negative triboelectric material (acrylic [73], PI [73], PDMS [192], silicones, PE [195], PVC [194], PTFE [191], FEP [44])	High output voltage and power [31] Available low-cost active materials Can non-contact sensing [72] Small output current

## Morphological Design of Sensing Materials

Several significant factors should be considered to develop sensing components with excellent and comprehensive sensing capabilities. Two crucial factors are functional materials with appropriate intrinsic properties [94, 95], morphological and geometric design of the sensing layer [77, 102]. Various functional materials with superior electronic properties, mechanical compliance, and large-area processing compatibility have been successfully used to fabricate flexible pressure sensors. In addition, many research groups have performed microengineering of the active layer to improve sensor performance [25, 64, 69, 71]. The active layer deforms under pressure, affecting the resistance [102], capacitance [64], piezoelectric [69], and frictional [71] electric output signals. Morphological and geometric microengineering of the active layer has been shown to improve performance parameters, such as the sensitivity, sensing range, limit of detection, and response/relaxation times. Table 2 compares the performance parameters of sensors based on different microstructures and sensing mechanisms. There are nanometer-scale or millimeter-scale structures due to the low weight and thinness of flexible pressure sensors. Some nanometer-scale pressure sensors utilizing band energy [94, 196], variable layer spacing [95–97], and cracks [98, 197] have been developed in recent years. And the commonly used microstructures include microroughness [93, 102, 103], porous hierarchical structure [32, 34, 37, 61, 104–106], and multiscale hierarchical structure [25, 63, 74, 82, 107–110]. In the following, we will discuss pressure sensors focused on these microstructures in detail.

**Table 2:** Summary of pressure sensors with their performance parameters

Key material	Type^a^	Sensitivity (kPa^−1^) and some output parameters^b^	Working range	Response/recovery time
rGO wrapped on electrospun P(VDF-TrFE) NFs [233]	R	15.6 kPa^−1^	1.2 Pa- 55 kPa	5 ms
Interlocking ZnO SUSM [74]	R	75–121 kPa^−1^ ( 0–200 Pa), > 15 kPa ^−1^ (200–10,000 Pa)	0.015 Pa-10 kPa	7 ms
MoS_2_-PDMS foam with leaf vein as Spacer [280]	R	150.27 kPa^−1^ (< 1 kPa), 1036.04 kPa^−1^ (1–23 kPa)	6.2 Pa- 23 kPa	50/40 ms
Sea urchin-like Fe_2_O_3_/C@SnO_2_ on the melamine sponge [78]	R	680 kPa^−1^	0.52 pa-150 kPa	10/22 ms
Three-layer stacked CNTs- and Ni-coated fabrics [50]	R	26.13 kPa^−1^	200 Pa-982 kPa	83/88 ms
SBS/Ag NPs with PDMS on kevlar fiber [65]	C	0.21 kPa^−1^ under 2 kPa, 0.064 kPa^−1^ above 2 kPa	8 mg- 3.9 MPa	40/10 ms
Micropyramidal SWNT /PDMS with Al_2_O_3_ dielectric layer [156]	C	0.7 kPa^−1^ up to 25 kPa	120 kPa	50 ms
Graded intrafillable architecture-based PVA/H_3_PO_4_ film [75]	C	220 kPa^−1^ (0.08 Pa-360 kPa)	0.08 Pa-360 kPa	9/18 ms
MXene and inoic membrane [157]	C	S_min_ > 200 kPa^−1^, S_max_ > 45,000 kPa^−1^	20 Pa- 1.4 MPa	98/70 ms
Microstructured PDMS with Pil_2_TSi semiconductor [164]	Tr	8.4 kPa^−1^	20 kPa	10 ms
Electrospinning Ag NF-Ag NW network electrode with SiO_2_ dielectric layer [48]	Tr	1.78 × 10^–3^ kPa^−1^ (< 350 kPa)	1.6 MPa	32/56 ms
Pyramidal PDMS/CNTs electrode with semiconductor MoS_2_ [279]	Tr	> 100 kPa ^−1^	600 Pa-130 kPa	50 ms
Sandwich-structured FEP/Ecoflex/FEP [2]	P	32.6 nA kPa^−1^ at 0.125–5.25 kPa, 6.71 nA kPa^-1^ at 7.5-22.5 kPa	125 Pa-22.5 kPa	18.6 ms
R layer: pyramid rGO array; P layer: P(VDF-TrFE) [186]	R & P	R:14.5 kPa^−1^ P:1.62 V kPa^−1^	R: 15 Pa-4 kPa P: 100 Pa-9 kPa & 0.05–700 Hz	
Micro-patterned PDMS-CNTs and PDMS [122]	Te	0.51 V/kPa, 0.7 µA kPa^−1^	5 kPa-450 kPa	
Wood [21]	Te	0.78 V/(m s^−1^) (< 4.5 m s^−1^), 0.21 V/(m s^−1^) (> 4.5 m s^−1^)		
Nylon fabric [31]	Te	7.84 mV Pa^−1^	Up to 20 Hz	20 ms
PTFE with porous networks of Ag NWs electrode [238]	Te	0.1 nA kPa^−1^, σ is 40 μA cm^−2^, V_oc_ is 14 V, I_sc_ is 0.28 μA		200 ms

^a^R: Piezoresistive, C: Capacitive, Tr: Transistor, P: Piezoelectric, Te: Triboelectric

^b^I_sc_: short-circuit current density, V_oc_: open-circuit voltage, σ: transfer charge density

### Nanometer-scale Structure

**Fig. 3:**
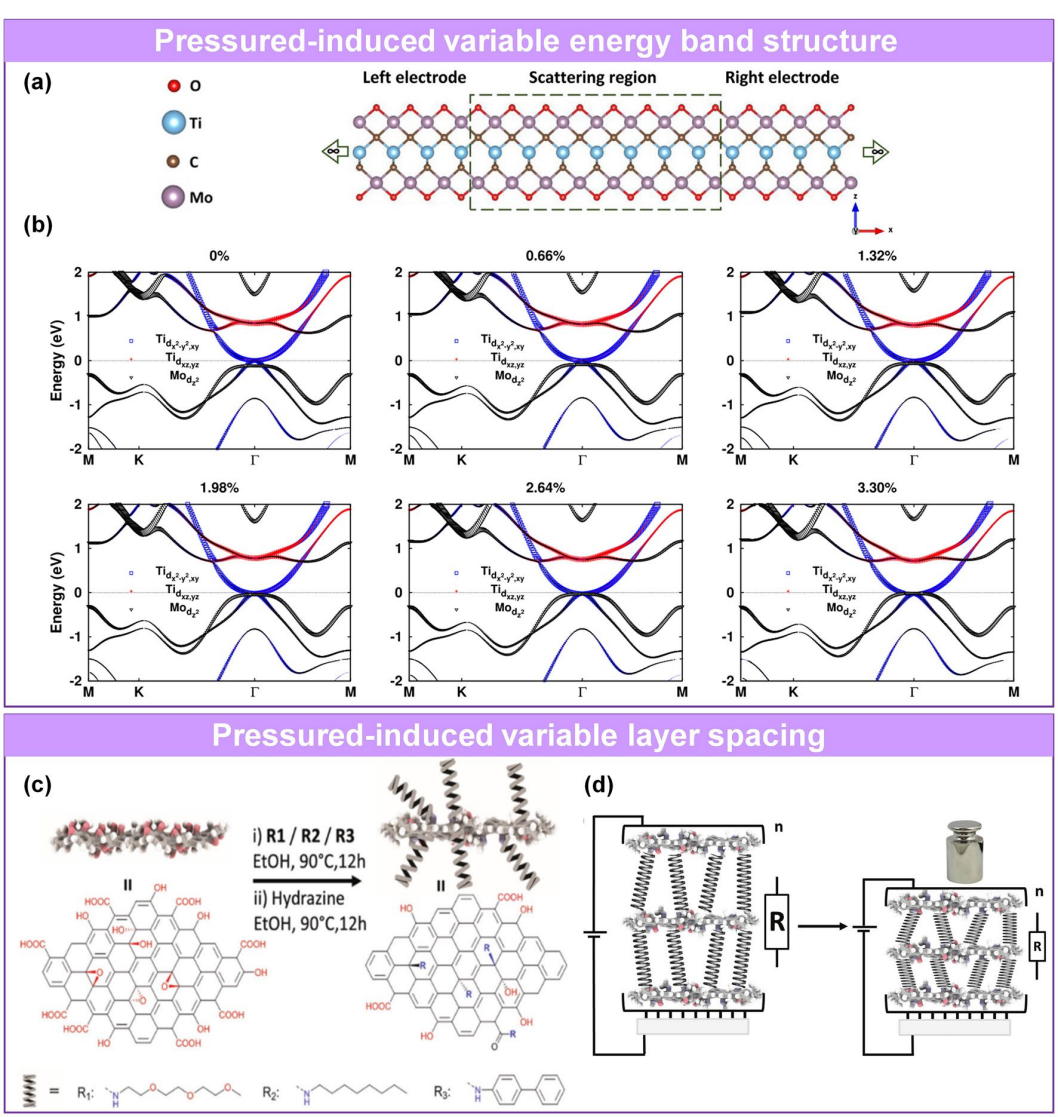
Sensing materials with nanometer-scale variable structures. **a** Model of Mo_2_TiC_2_O_2_ junction. **b** Electronic band structures of compressed Mo_2_TiC_2_O_2_, where the energy levels are indicated by the dashed lines [94]. Copyright (2020) Elsevier. **c** Schematic diagram of covalent connections between different rigid molecules (R_1_, R_2_, R_3_) and graphene. **d** Working principle of pressure sensor and contact variation of pressure unloading/loading [96]. Copyright (2018) Wiley–VCH

Some materials are suitable for constructing fine quantum-level intrinsic pressure sensors based on tunneling effect [139] or variable band structure [94, 196, 198–203] under pressure. Nayak et al. investigated the structural, vibrational, electrical, and optical dependence of single-crystalline MoS_2_ under pressures [196]. These results show a lattice distortion involving anisotropic *c/a* axial compression beginning at ~ 10 GPa in multilayered MoS_2_ leading to an intermediate state followed by a pressure-induced semiconducting to metallic transition at ~ 19 GPa, which can be attributed to sulfur–sulfur interactions as the van der Waals gap closes at high pressures. The results open a new window of opportunity for the development of nanoscale pressure sensors, switches, and multi-physics devices using multilayered MoS_2_ and semiconducting transition metal dichalcogenides. Similarly, Akkuş et al. reported a compressed double MXene (Mo_2_TiC_2_O_2_)-based pressure sensor with an electronic band structure that operates in the quantum conductance regime [94]. The electrons at the Fermi level of the double MXene originate from Ti and Mo atoms (Fig. 3a). The band structure is pushed upward under pressure, changing the electronic structure near the Fermi level. The frequency band related to Mo is affected more significantly. This process causes a significant change in the valence band maximum (VBM), changing the conductivity (Fig. 3b).

Pressure sensors with variable layer spacing of lamellar materials [95–97, 147] have also been proposed in recent years. Gao’s group fabricated a piezoresistive sensor based on multilayer Ti_3_C_2_T_x_ MXene [95]. The interlayer distance changed significantly under compression, as observed in the transmission electron microscopy (TEM) image. Huang et al. designed a piezoresistive sensor whose layer spacing was controlled by a molecular chain [96]. The three oligomers with different rigidity (poly(ethylene glycol) (R_1_), polyethylene (R_2_), and poly-(p-phenylene) (R_3_)) were covalently linked to the graphene basal plane through an epoxy ring-opening reaction (Fig. 3c-d). According to Hooke's law, the most elastic molecular grafted composites have the highest sensitivity, which was demonstrated in the experiment. Although the sensitivity, working range, and other sensing parameters of these pressure sensors are inferior to sensors with large structural changes, the novel principle provides guidance for the design and construction of sensors.

Additionally, constructing microcracks [98–100] is also a classic design strategy to obtain pressure sensors with high sensitivity. Although the design of microcracks is often used for the flexible tensile strain sensors, some of them can also be employed to monitor the stimulation of vibration and pressure [98, 99]. Inspired by the geometry of a spider’s slit organ, which has ultrahigh sensitivity, Kang et al. proposed a flexible sensor based on nanoscale crack junctions [98]. The disconnection–reconnection process of the zipper-like nanoscale crack junctions of the Pt films on polyurethane acrylate (PUA) under strain or vibration resulted in ultrahigh mechanical sensitivity of the sensor. The nanoscale crack junction-based sensory system could be useful in diverse applications requiring ultrahigh displacement sensitivity, such as sound and speech pattern recognition, human physiology monitoring, and flow rate indicators. A similar mechanical crack-based sensor with high sensitivity was proposed by Choi et al. based on the guided formation of straight mechanical cracks [99]. The sensor has patterned holes on the surface of the device to concentrate the stress near the holes, resulting in the formation of uniform cracks connecting the holes at the surface. The guided straight crack formation resulted in a sensitivity of over 1 × 10^5^ at pressures of 8–9.5 kPa.

### Micrometer-scale Structure

Micrometer-scale pressure sensors are the most commonly used microstructure of pressure sensors for both electrode [89, 90, 93, 147] and active layer [25, 48, 62, 75, 88]. Major structures include microrough structures [93, 102, 103], porous structures [32, 34, 37, 61, 104–106], and multiscale hierarchical structures [25, 50, 63, 74, 82, 107–110]. These are discussed in detail below.

#### Microrough Structure

Sensing materials with a microrough structure can be divided into microgeometric [75], wave [93], and wrinkle [103] structures. These microstructures can be constructed directly in active materials [93] or by depositing functional materials on rough flexible substrates (microstructured PDMS, PU, Ecoflex, rubber, etc.) [102].

A typical shape of these micropatterns is the pyramid, which was first proposed by the Bao’s group for capacitive pressure sensors in 2010 [163]. As the cones are deformed, they assume a more rectangular shape, increasing the contact area with the electrode. The advantage of the pyramid shape is the uneven stress distribution, with the maximum stress at the tip, resulting in larger changes in the contact area than in the unstructured surfaces under a given applied pressure. Due to advances in micropatterning technology, micro-geometric structures, such as micro-columns [90], micro-pyramids [148, 204], micro-domes [107, 205], micro-ridges [117], waves [93], wrinkles [103, 156], polylateral papillae [63], as well as fish scale surface [206], bionic micromorphology surface-by-surface treatment [93], duplicating form (such as leaf [207], petal [76], silk mold [208]) or other customized patterns [102, 148] have been constructed in piezoresistive [102], dielectric [75], piezoelectric [25], and triboelectric layers [71]. Microstructures with reduced modulus can improve deformation ability and achieve a timely response [209]; microstructures with a larger specific surface area can increase the deformation space and contact sites [74, 102]; microstructures with high transfer charge capacity [71, 88] can maximize the conduction of electrical signals. The above designs provide increased sensor performance, including high compressibility, high sensitivity, a low detection limit, and a fast response time.

**Fig. 4:**
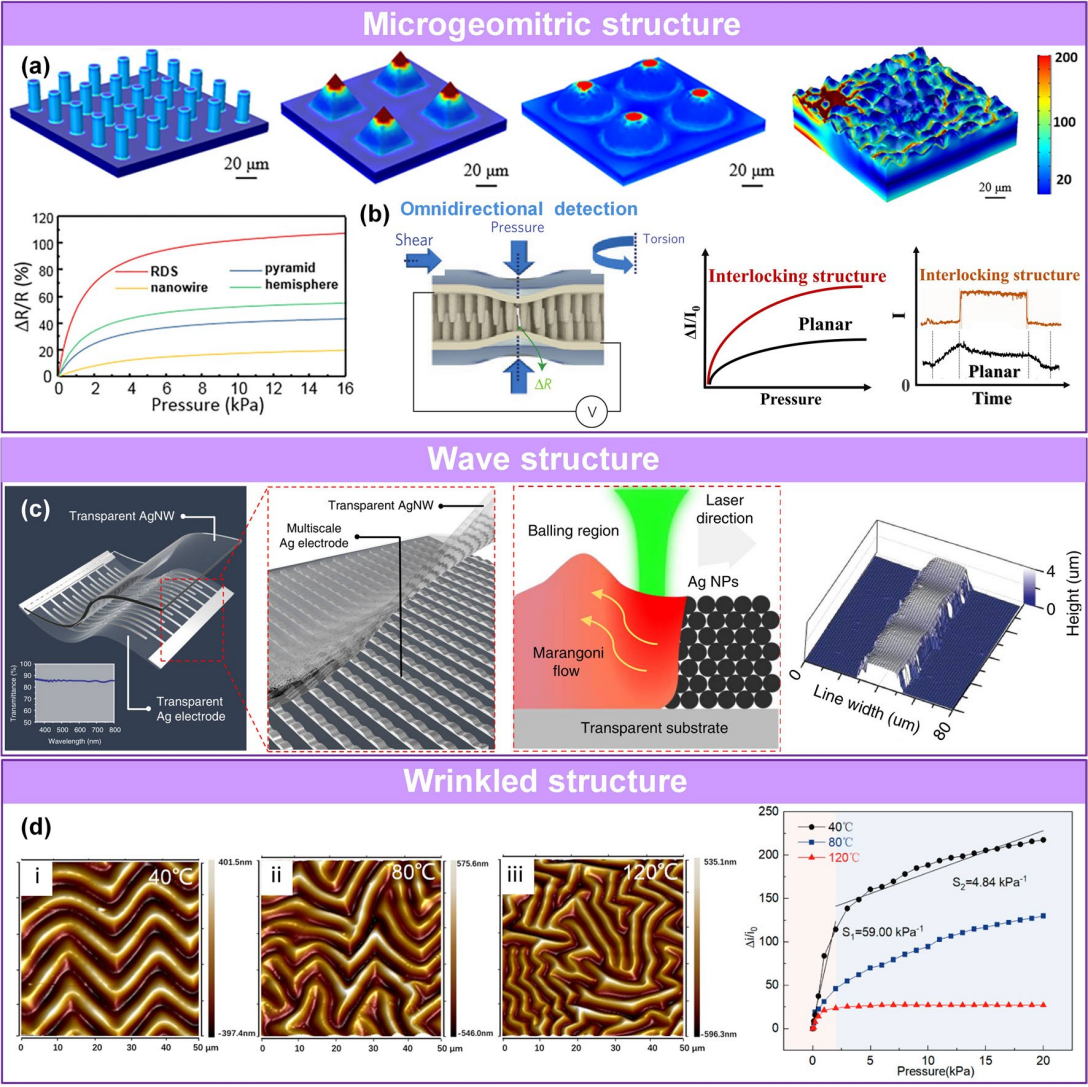
Sensing materials with microrough structures. **a** Sensing materials with microgeometric structures [102]. Copyright (2018) American Chemical Society. **b** Advantages of interlocking structures [25, 140]. Copyright (2015) Wiley–VCH and (2012) Springer Nature. **c** Wave structures [93]. Copyright (2019) The Authors. **d** Wrinkle structures [103]. Copyright (2021) American Chemical Society

Finite element analysis (FEA) has been widely used to optimize the sensing performance of various pressure sensors using COMSOL [70, 72, 75, 79, 102, 117] and Abaqus [65, 103]. Several representative microstructures have been investigated. Ren’s group simulated the stress distribution of microcolumn, pyramid, dome, and randomly distributed spinosum (RDS) micro-geometric structures under typical pressure values [102]. The microcolumn structure showed uniform pressure distribution in the height direction and the lowest sensitivity. The stress of the pyramid and micro-dome structure was concentrated at the top of the region, whereas that of the randomly distributed ridge structure was concentrated at the initial contact peak and the base of the adjacent peak due to stress transfer. This feature contributed to a more uniform stress distribution and resulted in higher yield strength and a larger linear range. Moreover, gaps remained after the contact between the concave and convex surfaces, which can further delay the saturation of contact. The size of the contact area of these structures followed the order microcolumn < pyramid < dome < RDS (Fig. 4a). Thus, the sensors with the RDS microstructure had the highest sensitivity and largest sensing range. Microgeometric structures are often assembled in the form of single-sided microstructures [75, 150] and double-faced interlocking microstructures [102, 148]. The biological system-inspired interlocking structures can realize some characteristics of the resultant sensors, such as various external stimulus perception [140], high sensitivity [148], a fast response [25] (Fig. 4b), and minimum mechanical damage [74, 78]. Pang et al. reported a highly flexible, multiplex pressure and strain sensor based on interlocking high-aspect-ratio Pt-coated polymeric nanofiber arrays that mimic the microstructures of beetle wing-locking [140]. Mechanical sensing was enabled by numerous tiny contacts between the neighboring high-aspect-ratio fibers on flexible supporting surfaces. The sensor was capable of omnidirectional detection of pressure, torsion, and shear force with high sensitivity.

Similarly, wave structure is also a typical microrough structure, which can be fabricated by templating, pre-straining [66, 109], solvent evaporating [109], and surface induction [93]. Selective laser sintering is a surface treatment technology that can be utilized to induce non-flat morphology, where the morphology is controllable by reasonably regulating the irradiation parameters. Ko’s team designed the regular wave structured electrode array, which are self-generated due to the action of surface reconstruction caused by the material of Ag NPs melting, convection flowing and solidifying under the given laser-induced thermal gradient (Fig. 4c) [93]. The first demonstration of a flexible and transparent sensor, which is capable of determining 3D information in a single device, can be realized through the above unique self-generated multiscale silver micro-pattern.

Wrinkles are a low-cost microrough structure that can be prepared at a large scale. Typically, wrinkles can be fabricated by thermal shrinkage polymer templating [63], changing the surface energy [210], doping, and embedding [156]. They have been introduced into active materials such as graphene [103], PPy [76], MXene [210], and CNTs [156]. Tang et al. used a thermal method at different annealing temperatures to create wrinkles with different wavelengths (λ) and amplitudes (A) (Fig. 4d) [103]. The formation of the wrinkles occurred due to the difference in the thermal expansion coefficient between graphene nanowalls (GNWs) and PDMS. Wrinkles formed when the maximum principal stress reached a critical value. The stress included the thermal residual stress and the compressive stress in the film during cooling. The morphology of the wrinkled GNWs was easily controlled by adjusting the thickness of the GNWs film and the annealing temperature. As the wavelength and amplitude height of the wrinkles increased, the level of deformation increased, enlarging the contact area and improving the sensor’s sensitivity.

#### Hierarchical Structure

Some structures are stacked or combined, forming a hierarchical structure to optimize the sensing performance. Typically, the commonly used hierarchical structures in the sensing layer of pressure sensors mainly include porous hierarchical structure and multiscale hierarchical structure.

**Fig. 5:**
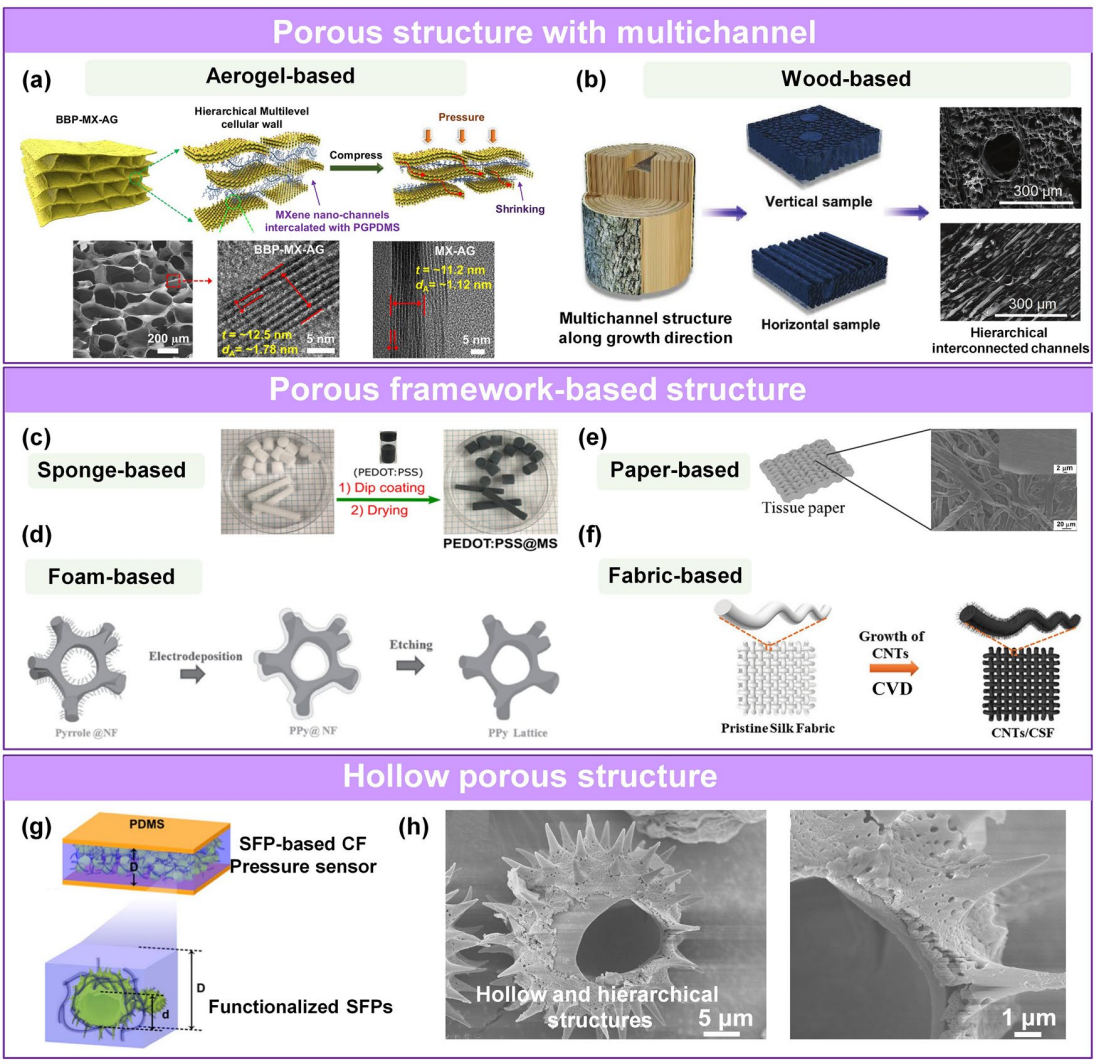
Sensing materials with porous hierarchical structures. **a** Aerogel-based [104]. Copyright (2022) The Authors. **b** Wood-based porous aerogel with nanochannels in the cellular walls [61]. Copyright (2018) Wiley–VCH. **c** Porous structures fabricated by coating and depositing of sponge-based [37] (Copyright (2018) American Chemical Society), **d** foam-based [105] (Copyright (2016) Wiley–VCH), **e** paper-based [34] (Copyright (2019) American Chemical Society), **f** fabric-based [106] (Copyright (2020) Wiley–VCH). **g** Piezoresistive sensor based on SFP-based CF. **h** SEM image of hollow hierarchical structure [225]. Copyright (2017) Elsevier

**Fig. 6:**
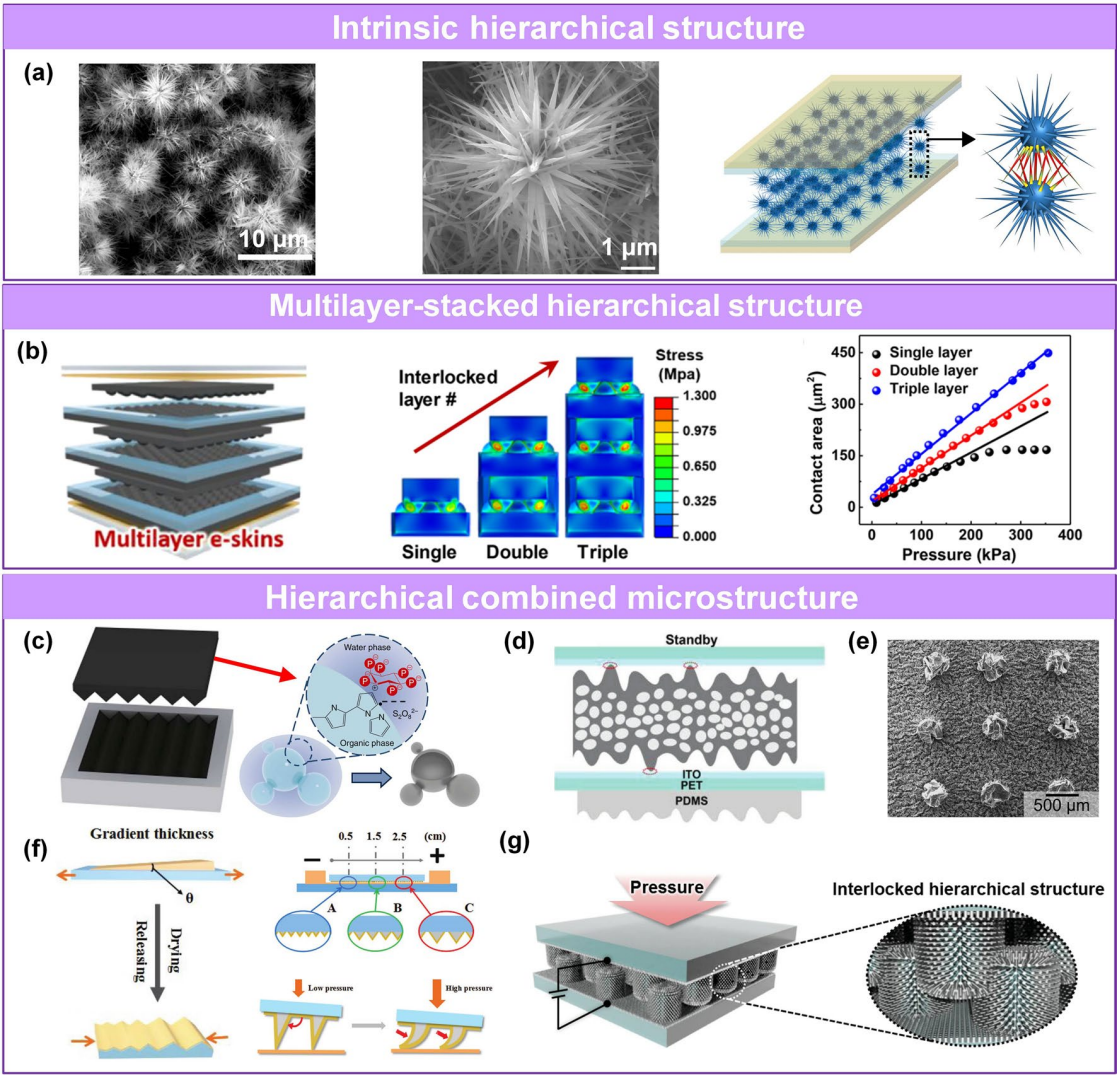
Sensing materials with multiscale hierarchical microstructures. **a** Intrinsic hierarchical structure with tapering spine [74]. Copyright (2018) The Authors. **b** Schematic diagram, pressure distribution simulation and sensing performance comparison of multilayer-stacked structure [107]. Copyright (2018) American Chemical Society. **c** Combined hierarchical structure of the hollow material is molded into a slightly rough surface [82]. Copyright (2014) Springer Nature. **d** Porous with microrough structure [108]. Copyright (2019) Wiley–VCH. **e** Wrinkles with microrough structure [63]. Copyright (2020) American Chemical Society. **f** Wrinkles with height gradient [109]. Copyright (2019) Wiley–VCH. **g** Interlocking structure combined with interlocking structure [25]. Copyright (2015) Wiley–VCH

1. Porous hierarchical structure Porous materials are popular for the construction of pressure sensors due to their large specific surface area, elastic reversibility, low density, and light weight. Interconnected frameworks like foam-, sponge-, aerogel-, paper-, and textile-based network structures, hollow structure are all porous structures. Considerable part of porous structure is actually kind of hierarchical structures and the pore usually has various multiscale structures. In addition, some pore wall of porous structure is also composed of active materials with microstructure, such as small pore structure [61], interlayer structure [104], wrinkle morphology [211], and network structure [212], so we locate the classical porous structure part in the hierarchical structure section. Sensors with porous structure are suitable for the fabrication of flexible and portable electronic devices [126]. In addition, their unique structure modulates the compressibility of the active layer and provides abundant contact sites under external loading, improving the performance of the sensors. The main advantage of the porous layer in capacitive pressure sensors is the incorporation of air holes with lesser deformation resistance and larger capacitance of the dielectric layer since the separated conductive matters can equate to numerous electrodes of decreased electrodes plates distance [64]. As a result, the capacitance and sensitivity are high. A porous structure is used in resistive and triboelectric sensors to increase the contact area between the two surfaces to effectively change the conductive path [61, 86, 87] or enhance the triboelectric effect [73, 213], respectively.
  Aerogel is a commonly used porous structure [62, 113, 142, 214]. Active aerogel with multichannel structure can be spontaneously generated using specific synthesis technologies, such as a microfluidic method [84], self-assembly [142], and self-foaming combined with freeze-drying [27, 64]. Liang’s group reported a multiscale aerogel with nanochannels inside the cellular walls (Fig. 5a) [104]. In this work, the soft bottlebrush polysiloxane was intercalated into MXene interlayers via covalent crosslinking, and the obtained composite material with interlayer structure is further frozen and dried to form porous aerogels. Under pressure, the porous structure of the active material and the layer spacing in the cell walls change simultaneously, resulting in an ultrahigh sensitivity of the corresponding sensor. Natural wood possesses a distinctive 3D microstructure containing hierarchical interconnected channels along its growth direction. Huang et al. fabricated a sensor based on carbonized wood filled with PDMS (Fig. 5b) [61]. The unique multichannel is analyzed by comparing the pressure response of the sensors prepared by vertically cut and horizontally cut composite. The results show that the sensors made of horizontally cut composite exhibit much higher sensitivity (10.74 kPa^−1^) and wider linear region (100 kPa, R^2^ = 99%) due to their rough surface and largely deformable microstructure. Additionally, the sensor also shows little hysteresis and good cycle stability, which can accurately monitor human pulse and detect respiration rate.
  Additionally, some porous materials with interconnected framework, such as foam [215], sponge [37], textile [50, 130, 216], and paper [28, 34, 110] (Fig. 5c-f), are suitable as elastic skeletons for loading the sensing material by impregnation [28, 37, 85, 217], deposition [105, 106, 218], or being directly carbonized to get active layers [110, 134]. Typically, in existing works, porous foam and sponge were dip-coated with micro-/nanoscale functional materials to prepare porous sensing layer, such as CNT/TPU/silicone foam [219], (multiwalled carbon nanotubes) MWNT/(reduced graphene oxide) rGO/PU foam [217], PEDOT:PSS@melamine sponge [37], PDMS/Ag NP sponge [220], metal-coated PDMS sponge [221], PDMS/CNT sponge [222], CB@PU sponge [223], PU/graphene sponge [87]. In addition, electropolymerizing and sputtering were employed to prepare porous sensing materials like PPy foam [105], CNT-based fabric [106], and Au@PU sponge [218]. Ding et al. prepared a piezoresistive pressure sensor based on PEDOT:PSS@melamine by one-step dip coating the commercial melamine sponge in an aqueous dispersion of PEDOT:PSS. The resultant sensor exhibited excellent compressibility and cyclic stability [37]. Li’s group designed a 3D PPy@graphene/PDMS sensing foam [105], whose manufacturing process includes the fabrication of PPy foam (electropolymerizing pyrrole onto nickel foam followed by the etching of the substrate), dip-coating of graphene, and infiltrating of PDMS. Under compression, the sensing materials with elastic porous frame show excellent cycling durability.
  The development of fabric-based and paper-based sensing layer like graphene/paper [28], Au NW/paper [138], MXene/paper [34], rGO-Ag NW@cotton fiber [85], and MXene-textile [130] further enriched the design and application of pressure sensors. Gong et al. developed an efficient strategy for constructing highly sensitive and flexible piezoresistive pressure sensors by sandwiching ultrathin Au NW-impregnated tissue paper between two thin PDMS sheets [138]. Tao et al. proposed a paper-based pressure sensor by integrating tissue paper with graphene [28]. They found that the number of tissue paper layers significantly affected the sensitivity of the pressure sensor. The multilayer paper-based graphene pressure sensor exhibited stable performance in the range of 0–20 kPa and high sensitivity of 17.2 kPa^−1^ (0–2 kPa) due to the existence of air gaps. And it is worth noting that textile-based composite sensing materials are well suited for fabricating wearable pressure sensors due to the scalability of the fabrication method [28, 130], its light weight, good mechanical properties, breathability, soft and high wearing comfort. Furthermore, with the development of materials and technology, various water-resistant devices have been successfully prepared. Gogotsi’s group reported washable MXene-coated cellulose yarns textile-based capacitive pressure sensor, where this sensor can tolerate 45 cycle washings at temperatures of 30–80 °C [224]. In addition, in this work, they introduced different weaving methods (single jersey, half-gauge, interlocking structure). Wang and co-workers fabricated a machine-knitted washable textile TENG, which can still precisely monitor physiological signals after several times of washing [31].
  Moreover, hollow materials with common high compression resilience have also been widely used to construct pressure sensors [32, 69, 113]. The hollow hierarchical structure with enhanced contact change and deformation capability contributes to an excellent sensitivity and cycle performance. Cho’s group designed a piezoresistive sensor based on CNT-functionalized sunflower pollen (SFP) microcapsules composite film (CF) (Fig. 5g-h) [225]. The resultant capsules with hollow and durable sporopollenin biopolymer wall are highly elastic and can sustain large mechanical deformations. Under pressure, the microstructure frame (D→D’) and functionalized SFP microcapsules (d→d’) deformed readily, and the hierarchical structure with interlocking contact lead to an effective electrical conductance change through the sensing layer, resulting in high sensitivity of the corresponding sensor.
2. Multiscale hierarchical structure The multiscale hierarchical structures used for pressure sensors mainly include intrinsic hierarchical structure [74], multilayer-stacked hierarchical structure [50, 107], and the combined hierarchical structure made up of multiple single structures [25, 63, 82, 108, 109]. Some intrinsic hierarchical structure has excellent stress concentration effect [74]. Inspired by organelles with excellent signal transduction ability, Yin et al. synthesized zinc oxide sea urchin-shaped microparticles (ZnO SUSM) with an intrinsic hierarchical structure and a tapering spine (Fig. 6a) [74]. The particles were fabricated into a sensing film via droplet casting. The resulting sensor had an ultralow detection limit of 0.015 Pa and could monitor mass changes as a drop of 40 μL ethanol evaporated.
  Typically, a multilayered structure is always based on textile materials [50] or other types of interlocking structures [107]. Lee et al. fabricated a multilayer-stacked interlocking structure, whose contact area increased under pressure to realize the detection of pressure (Fig. 6b) [107]. A multilayered structure improves the stress distribution of each layer and increases the linearity range. It is worth noting that the number of conductive paths increases initially with the increase of sensing layers or layer thickness, resulting in high variability of the electrical signals under pressure. However, a further increase in the number of sensing layers or layer thickness will lead to the decrease of variable electrical signals under pressure and a poor sensor performance.
  Several combined hierarchical structures were developed to improve the sensing performance of pressure sensors, including a porous structure with microgeometric surface morphology [121], porous structure with microrough surface [108], wrinkles with a microgeometric structure [63], and wrinkles with a height gradient [109], and microgeometric structure with interlocking microstructure [25] (Fig. 6c-g). These designs endowed the pressure sensor with high sensitivity, wide line range, a low detection limit, and fast response/recovery. Bao’s group developed a hollow spherical PPy hydrogel using a multiphase aqueous solution reaction, where the hollow spherical hydrogel was further molded into microrough surface structure for the large contact variation under pressure (Fig. 6c) [82]. Ha et al. developed highly sensitive and rapid response e-skins with an interlocking, hierarchical micro/nanostructures, consisting of PDMS micropillars decorated with ZnO NWs arrays (Fig. 6g) [25]. The interlocking system led to a significant increase in the effective contact area between the NWs in response to external stimuli, resulting in highly sensitive piezoresistive static sensors. The sensor was also suitable for detecting dynamic tactile signals due to the piezoelectric properties of the ZnO NWs.
  In recent years, there has been increased interest in micropatterning the electrode instead of the active layer. Several structures have been used, including 3D micro-column electrode [89, 90], micro-pyramid [92] and lamellar structure with variable layer engineering [147]. The rationale behind this design is analogous to micropatterning the active layer. Moreover, microstructured electrodes have been combined with the structured sensing layer to optimize the sensing performance in some research work [89]. These all highlights show the important role of structural engineering.
  Considering the wide range of variable regulation of each microstructure, it is difficult to identify the complex effects of each micromorphology for unified comparison. Nevertheless, some general conclusions still can be drawn. The nanometer-scale interlayer structures’ engineering is inferior to those microstructures with micrometer scale to get good sensing performance, but the novel mechanism or design still inspires researchers to conduct in-depth research. Microrough structures with the characteristics of abundant contact variation are very suitable for piezoresistive sensor to achieve high sensitivity, but the corresponding working range is often limited. Porous structures with excellent compression are suitable for achieving high sensitivity and stability, while the hierarchical structures with the improved deformation ability and increased deformation space is conductive to a timely response, high sensitivity and wide sensing range. These features of microstructures are summarized in Table 3.

**Table 3:** Characteristics of different microstructures in flexible pressure sensors

Micromorphology	Characteristics
Interlayer microstructures	Precise control over the adjustment of the material structure [96] An excellent stability due to the intrinsic property of sensing materials The sensitivity and sensing range are relatively limited [95]
Microrough microstructures	Lead to an increased specific surface area [102] The extensive contact changes can lead to high sensitivity, especially suitable for piezoresistive and triboelectric pressure sensing [129] The size of related device is apt to achieve extremely thin The compressibility is inferior to that of porous structure
Porous microstructures	Possess large compressibility with significantly reduced modulus and stronger deformation ability With a low LOD due to the low density and good deformation ability [77] Can result in a remarkable change in plate spacing and high sensitivity for capacitive pressure sensor [64] Some porous skeletons involving polymers makes the response time to stability relatively long
Multiscale hierarchical microstructures	With more deformable space and enhanced the deformation ability conductive to a high sensitivity and wide linear range [107] With a significantly reduced LOD and high sensitivity [74] Good bonding strength between different microstructures is necessary

## Microstructure Pressure Sensors with Superior Properties

Many important parameters have been used to evaluate the sensing performance of pressure sensors, including sensitivity (S) [75], limit of detection (LOD) [74], working range [79], degree of hysteresis (DH) [84], and sensing directionality [226, 227]. In addition to selecting sensing materials with excellent intrinsic characteristics [228], the microengineering of the active layer is vital to achieving high performance of sensing devices. As mentioned in the previous part, many achievements have been made in the microengineering of the sensing layer, including microrough surfaces and hierarchical structures. However, it is noteworthy that not all performance parameters can be optimized simultaneously due to substantial differences in the size, feature spacing, and materials of pressure sensors. Although it is challenging, we explore the relationship between the microstructure design and the sensing performance of pressure sensors in this section. We discuss recent developments in the fabrication of sensors with high sensing performance.

### Pressure Sensors with High Sensitivity

The sensitivity of pressure sensors is a key index. It is defined as the ratio of the relative change in the output ($\Delta x/x$) to the change in applied pressure ($\delta P$), which can be expressed as follows:

3 $$S = \frac{{\delta \left( {\Delta x/x_{0} } \right)}}{\delta P}$$

where the *x* is resistance (*R*), capacitance (*C*), current (*I*) or voltage (*U*). High sensitivity is related to a high signal-to-noise ratio (SNR) [25], enabling the sensor to distinguish subtle changes in pressure. In addition, a greater sensitivity generally means that the material exhibits a significant structural change as a result of a small change in pressure [74]. Since the LOD is inversely proportional to sensor sensitivity in the most cases, a pressure sensor with a low LOD can detect more subtle signals. Thus, pressure sensors with high sensitivity and a low LOD are required to detect small changes in pressure.

**Fig. 7:**
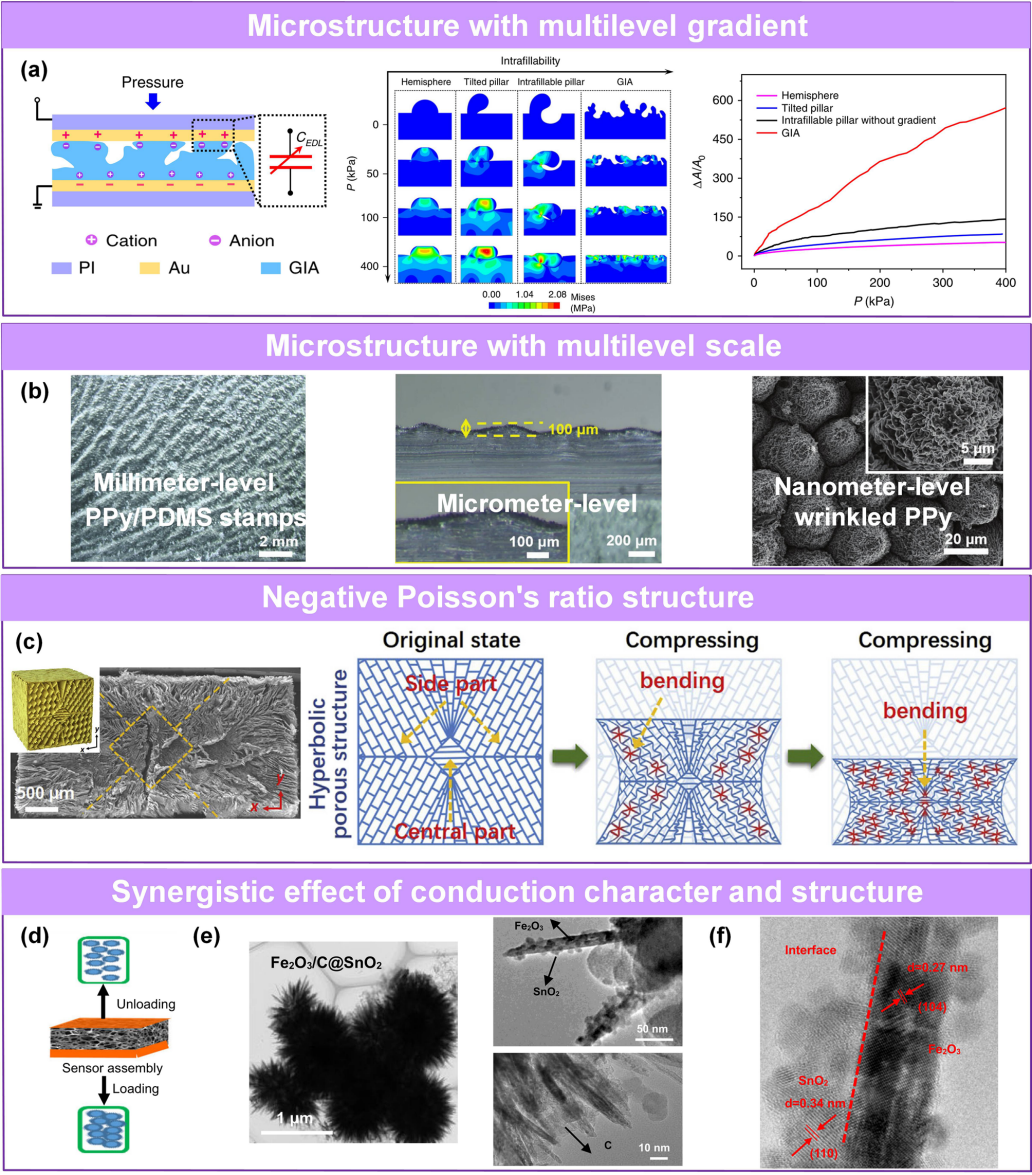
Pressure sensors with high sensitivity. **a** Schematic diagram, comparison of compression simulation and sensing performance of graded intrafillable microstructured ultra-capacitive pressure sensor [75]. Copyright (2020) The Authors. **b** PPy/PDMS stamps with millimeter/micro/nanostructures [76]. Copyright (2020) Wiley–VCH. **c** Negative Poisson's ratio structure with more contact under compression [77]. Copyright (2022) Elsevier. **d** Pressure sensor based on spongy skeleton combined with sea-urchin shape particles (SUSP). **e** TEM image and **f** high-resolution TEM image of heterogeneous interface in SUSP [78]. Copyright (2021) The Authors

As mentioned earlier, incorporating cracks is a classic design strategy to obtain high sensitivity of piezoresistive pressure sensors because it causes high variability in resistance. However, the effective range of such sensors is relatively small. Researchers fueled the development of ultrasensitive pressure sensors by incorporating microengineered sensing layers with numerous contact sites, good mechanical properties, and high compressibility. These sensors have high capacitance, resistance change, enhanced triboelectric effect, and high sensitivity. Bai et al. reported a microstructure iontronic piezocapacitive sensor with high sensitivity of S > 220 kPa^−1^ and a broad working range of 0.08 Pa–360 kPa [75]. In this work, the protrusions fabricated by duplicating sandpaper micro-rough structure can be bent and filled into concave parts upon compression, and the remaining gaps will become dense under further high pressures. This process substantially increases the contact area and significantly improves the specific capacitance due to the aggregation of positive and negative charges at the electron double layers’ (EDLs) interface under the applied voltage (Fig. 7a).

Multilevel-scale microstructure is obvious hierarchical structure contact. Yu et al. reported a pressure sensor based on a wrinkled PPy film/rose petal-shaped PDMS with a multiscale structure (millimeter/micrometer/nanometer) [76]. Its sensitivity was substantially higher than that of planar PPy film (70 vs*.* 2.62 kPa^−1^ at < 0.5 kPa) (Fig. 7b). Further, scholars utilize synergistic effects between material conduction character and material structure to improve sensitivity.

The positive Poisson's behavior of transverse expansion of conductive porous materials always inhibits their sensing performance. Liang’s group proposed a universal strategy for the fabrication of hyperbolic microstructure with negative Poisson's ratio effect based on a directional freeze-drying method (Fig. 7c) [77]. When the materials with this series of structures are compressed, the transverse and longitudinal shrinkage occur at the same time. This negative Poisson's ratio behavior can greatly increase the conductive pathway under compression due to the simultaneous contraction in transverse and longitudinal direction and thus can significantly improve the sensitivity of pressure sensor. Therefore, the pressure sensing sensitivity of porous MXene with negative Poisson's ratio can reach 990 kPa^−1^. Furthermore, the negative Poisson's ratio effect will lead to a denser structure under pressure and enhance the mechanical reliability of this porous metamaterial.

By leveraging synergistic effect of conductivity and structural characteristics of sensing materials, Wong’s group proposed a sea urchin-shaped microparticle-based high-sensitivity piezoresistive sensor with three heterojunction systems (Fe_2_O_3_/C, Fe_2_O_3_/SnO_2_, SnO_2_@C) on melamine sponge skeleton (Fig. 7d-f) [78]. In this work, apart from large current change under pressure at the semiconductor/conductor heterojunction interface, the (large-sized porous skeleton loading with hierarchical structured conductive loads) structure-induced contact change is the leading factor for pressure sensing. The tapering spines endow effectively mutual contact and promote signal conduction for low pressure; the porous sponge framework contributes to huge contact variation and wide sensing range.

### Pressure Sensors with a Large Working Range

**Fig. 8:**
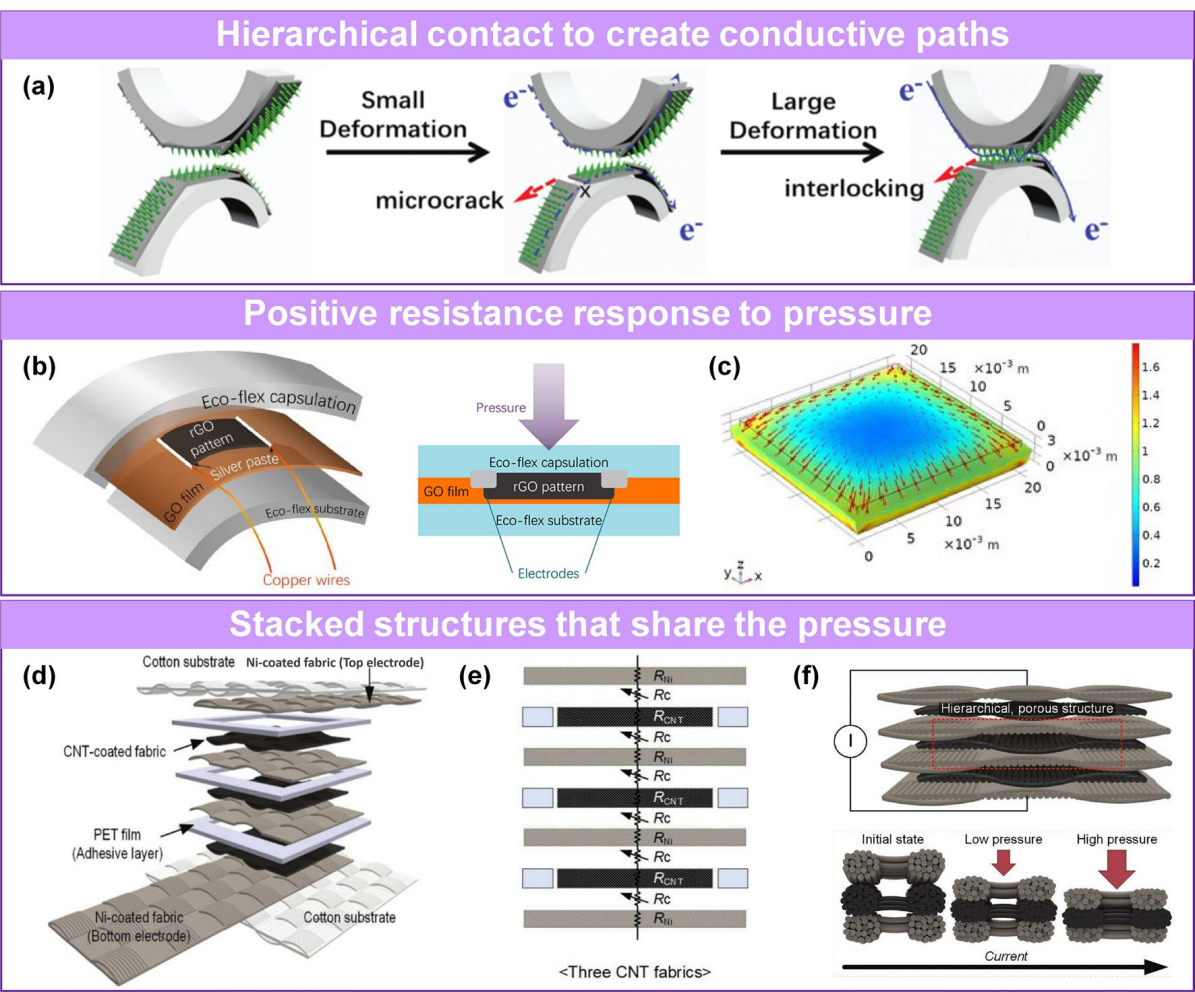
Pressure sensors with broad working range and stable sensing performance. **a** Schematic illustration of pressure sensor with microcrack and interlocking structure [35]. Copyright (2018) Wiley–VCH. **b** Device structure and **c** compression simulation of positive resistance variation [79]. Copyright (2020) American Chemical Society. **d** Schematic illustration, **e–f** Circuit diagram and sensing mechanism of the multilayer-stacked conductive fabric [50]. Copyright (2019) Wiley–VCH

Pressure sensors with a wide working range and long-term durability are required in many practical applications. Some pressure sensors lose their sensing ability due to contact saturation [229] or are damaged [74] under high-pressure loading. Thus, researchers developed numerous designs to address this problem. Guo et al. proposed that those active material systems that trend to be destroyed at high pressure can be optimized by introducing new contact conductive paths for a broad working range [35]. They designed a pressure sensor that incorporated microcracks and interlocking. Aniline coating and in situ polymerization were used to form interlocking hair arrays on rGO layers, utilizing the porous PU sponge backbone. Under compression, cracks appear in the rGO layer, changing the conductivity of the sensing material. The interlocking structures of the polyaniline nanohair (PANIH) connect as increasing the compression intensity, which creates additional conducting paths and ensures normal working conditions within high pressure (Fig. 8a).

In order to solve the problem of no more conductive paths caused by sensing material compression contact saturation [229] under high pressure, Ren’s group fabricated a positive resistance response pressure sensor with a wide working range by reasonably designing the compression structure with relatively low deformation in the center than the outer frame, which better protects the inner graphene (Fig. 8b-c) [79]. When pressure was applied in the z-direction, several kilopascals of pressure was insufficient to decrease the layer spacing of the graphene and boost the conductivity of the system. In contrast, the Eco-flex encapsulation layer caused the formation of random micro-cracks in the internal graphene film, substantially increasing the resistance of the sensor and its sensitivity.

In addition, a number of multilayer-stacked sensing materials have also been widely used in the construction of high sensitivity and wide range pressure sensors. Pyo et al. reported a multilayered Ni/CNTs/Ni fabric-based tactile sensor (Fig. 8d) [50]. The hierarchical structure of the fabric significantly increased deformation and contact change space (Fig. 8e-f). Additionally, the multilayered structure distributed the stress to each layer, resulting in high sensitivity (26.13 kPa^−1^) of the sensor in a wide pressure range (0.2–982 kPa). Although the sensitivity of a pressure sensor can be improved by increasing the number of sensing layers, a larger number of layers do not necessarily translate into better sensing performance. Too many layers result in an unstable signal; thus, three layers were used in this system.

### Pressure Sensors with Stable Sensing Performance

For pressure sensors, the stable sensing performance is very important for its practical application. Typically, the stability of pressure sensor mainly includes cyclic stability, storage stability and the stability toward anti-environmental interference. The cyclic stability is related to the compressive resilience of active materials [62] and the interfacial adhesion stability between electrode and sensing layer [81], while the storage stability of devices is usually concerned with the stability of active materials in the environment and the effect of encapsulation. There are many interferences in environment (e.g., dynamic temperature) affecting the sensing signals of the device, resulting in a diverse coupling results, and the accuracy of sensing. Anti-environmental interference stability means that the device can show stable sensing performance in variable environment. Among them, good storage stability can be achieved by selecting stable materials and effective packaging to block water and oxygen in the environment. For cyclic stability and anti-environmental interference stability, some interface engineering and dexterous microstructures were conducted to enhance the compressive resilience of active materials, stabilize the interface, and reduce these deformation caused by anti-environmental interference [25, 82, 83].

For example, although MXene has excellent electrical conductivity and water dispersibility [230], it is not possible to form compress resilient aerogel because it can only assemble into a random and loosely connected structure but not a continuous and ordered structure during the phase separation from the solvent [210]. Gao’s group mixed MXene with graphene oxide (GO) solution and prepared a compressible sensing aerogel via freezing assembly and lyophilization [62]. Graphene-functionalized MXene solves the assembly difficulties caused by the weak interaction of MXene interflakes, resulting in a complete three-dimensional porous frame with high compactness and toughness through Ti–O-C covalent bonding [231]. In addition, the cyclic stability of the resulting sensor is largely enhanced through the optimization of compressibility of the active materials.

**Fig. 9:**
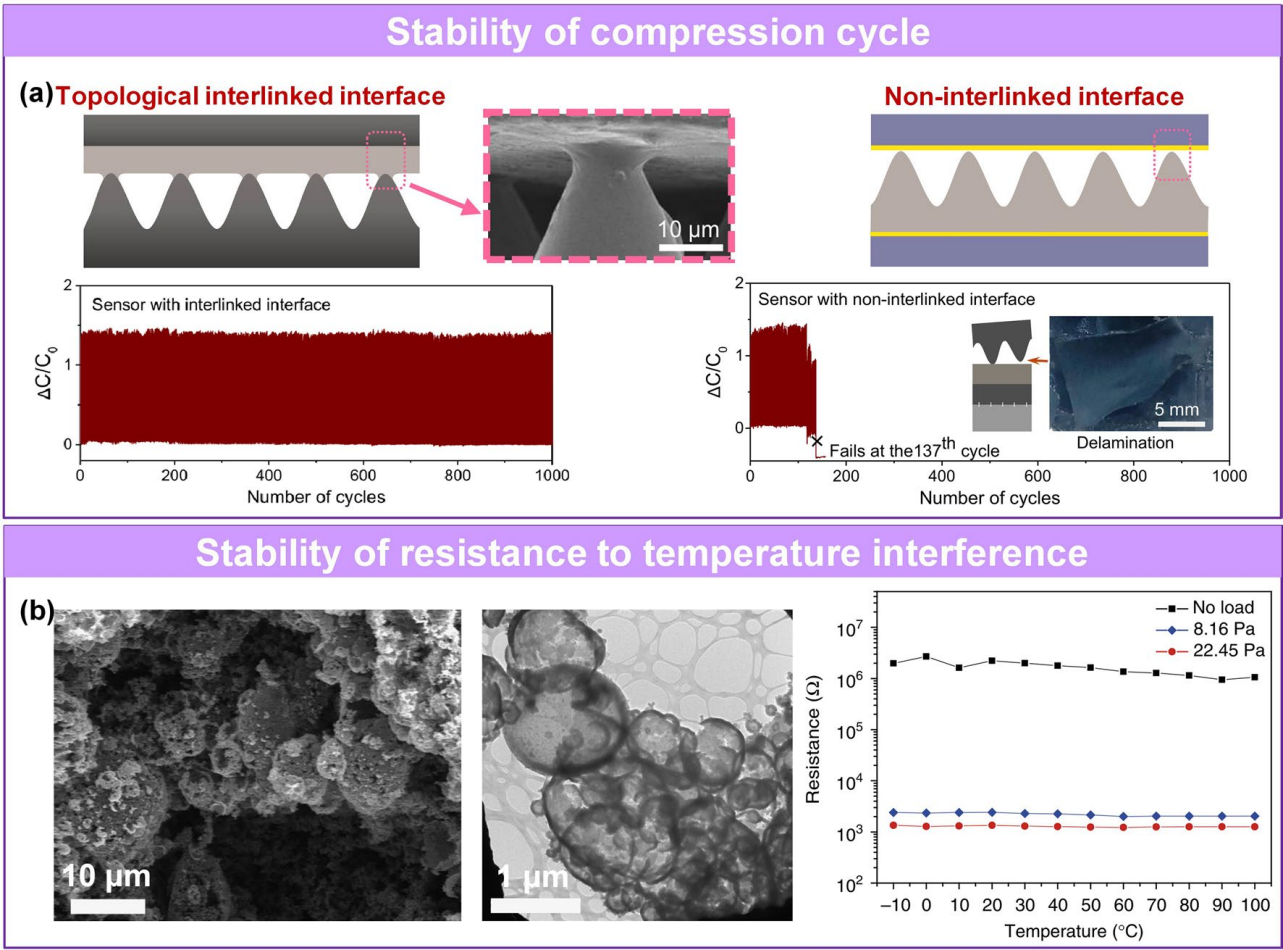
Pressure sensors with stable response. **a** Comparison of stable cycle performance of that with strong linked-interface and that with uninterlinked-interface [81]. Copyright (2022) The Authors. **b** Stable anti-temperature interference ability caused by hollow structure [82]. Copyright (2014) Springer Nature

Some researchers enhanced the wetting behavior of the conductive coating on the hydrophobic microstructured substrate to improve the stability of the device. The ethanol solvent-based PEDOT: PSS solution fabricated by solvent exchange has a better coating performance than the water-based PEDOT: PSS solution without affecting the inherent performance of PEDOT: PSS. The sensor with a uniform and conformal nano-coating film on the surface of the hydrophobic pyramid PDMS has more than 10,000 stable cycles [80]. Furthermore, as for the connection interface of active layer, Zhang et al. designed and compared the stability of the sensor with and without connection interface [81]. The results show that the sensor with interlinked interface possesses a more stable and repeatable output electrical signal than the counterpart (Fig. 9a).

Considering the thermal expansion of polymers, researchers have designed some temperature-stable pressure sensor based on the microstructure that can effectively reduce thermal expansion/contraction by relieving the thermal stress during temperature changes [82, 83]. Bao’s group designed a pressure sensor with the hollow-sphere microstructure, where the sensor at various ambient temperatures (from -10 to 110 ℃) exhibits almost the same electrical signal value under specific loadings (Fig. 9b) [82]. The hollow-sphere structure enabled the PPy to elastically deform, which also promote the contact stability of the pressure sensor and endow the device with stable and reproducible sensing performance. Moreover, they pointed out that crosslinking would further promote a stable sensing because each unit interacts with multiple polymerization chain. Similarly, the urchin-like hollow carbon spheres fabricated by Wu’s team show a temperature noninterference from 25 to 160 ℃ [83]

### Pressure Sensors with a Fast Response and Low Hysteresis

Real-time monitoring requires a fast response/recovery speed and low hysteresis of the pressure sensor. The viscoelasticity of the substrate/elastic skeleton [84, 153] and the interaction [232] between active conductive materials and the matrix are potential factors causing a response delay and high hysteresis. Recent works have focused on the following two aspects to solving these problems: (1) Optimizing the mechanical properties of the active layer by selecting materials with a suitable modulus, rapid deformation capability, low viscoelasticity, and strong bonding between the active material and the skeleton to prevent relative sliding and displacement. (2) Microengineering the active layer to improve the compressibility and elasticity of the material.

**Fig. 10:**
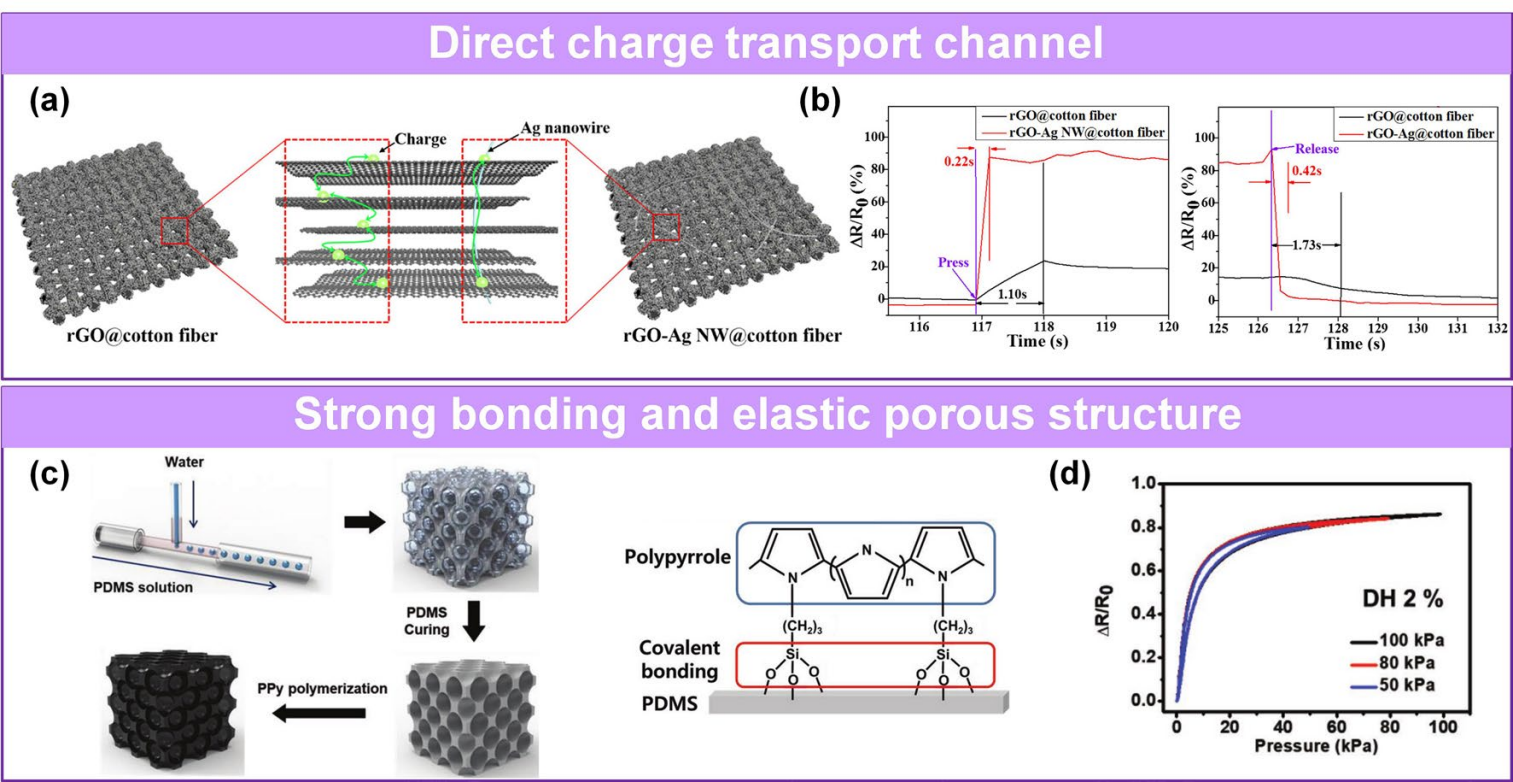
Pressure sensors with fast response and low hysteresis. **a** The mechanism diagram of charge transport and **b** response time comparison of rGO@cotton fiber and rGO-Ag NWs@cotton fiber [85]. Copyright (2018) Elsevier. **c** Fabrication process of and interaction between PPy and PDMS. **d** Hysteresis loops of sensor [84]. Copyright (2019) Wiley–VCH

Cao et al. designed a piezoresistive sensor with fast response/recovery speed based on rGO-Ag NWs@cotton fiber (Fig. 10a) [85]. The combination of the Ag NWs and the cotton fibers bridged the unconnected parts and cracks and provided more and faster conductive paths. The sensor exhibited response/recovery times from 1.10/1.73 s to 0.22/0.42 s (Fig. 10b). Cheng et al. designed a pressure sensor based on elastic crystal Si NWs [232]. The small contact area of the needle-like structures resulted in low interface adhesion, a low binding force, and hysteresis of 2.26%, with a fast response time of 3 ms. In addition, increasing the porosity of the active layer will improve the material’s deformability and recovery rate. Oh et al. fabricated porous PDMS through microfluidic technology to improve the compression resilience and incorporated conductive polymers PPy, which formed a strong covalent bond with the PDMS matrix, reducing the hysteresis to 2% (Fig. 10c-d) [84]. The equation for DH is as follows:

4 $$DH = \frac{{A_{unloading} - A_{loading} }}{{A_{loading} }} \times 100\%$$

There, the *A*_*loading*_ and *A*_*unloading*_ are the area of loading and unloading under the curve of ΔR/R_0_-P, respectively. Currently, resistive pressure sensors with porous structure [27, 233], single-faced micro-roughness [150, 234] and double-faced interlocking structure [102, 148] have exhibited ms-level response and recovery times. And a variety of microstructures have been utilized in capacitive pressure sensors to achieve a fast response time in the ms range, including randomly distributed microridges [75] and porous pyramids [121]. In summary, microengineering the active layer of pressure sensors is crucial for improving the response and recovery times. Common strategies for obtaining pressure sensors with superior properties, such as high sensitivity, broad working range, stable sensing, fast response, and low hysteresis, are summarized in Table 4.

**Table 4:** Strategies for some tiptop properties of pressure sensor

Object	Approaches
High sensitivity	Apply microstructured sensing material and electrode in pressure sensor [89] Introduce active material with super conductive and structural characteristic [78] Design multiscale structure to maximize the compressive contact under loading [77]
Broad working range	Design structure that can form new conducting path after being destroyed for normal sensing [35] Using sensing materials with hierarchical structure to increase contact change space [50]
Stable sensing	Establish a strong connection between the sensing layers and the connection between the sensing layer and the electrode [81] Design structures and materials with small thermal expansion effect [82]
Fast response and low hysteresis	Construct faster conductive channel [85] Design materials with rapid deformation capability and low viscoelasticity [84]

### Pressure Sensors with Other Characteristics

Pressure sensors with novel characteristics, such as high transparency [83, 226, 235, 236] and selective sensing [226, 227], have also been successfully implemented by microengineering the active layer.

#### Pressure Sensors with High Transparency

Pressure sensors with high transparency can be easily integrated into functional electronic devices to provide a clear view without affecting other units, which are well suited for human–computer interfaces and optical devices [93]. In addition, the design of transparent pressure sensor makes it possible to provide invisible camouflage electronic skin for imperceptible robot and prosthetics by integrating actuators with sensors [237]. However, the fact that many conductive carbon materials and their composites are not highly transparent is a big obstacle to developing pressure sensors suitable for medical image applications, e-skin, and touch screens. Some sensing systems have been successfully made transparent by microengineering the active layer to obtain a suitable layer thickness and refractive index with a minimum amount of materials.

A thin network can be prepared by electrospinning, and the density and effective thickness can be controlled by adjusting the electrospinning parameters, such as the concentration of the electro-spun materials and the electrospinning time [48]. Someya’s group fabricated a transparent pressure sensor based on the ultrathin electro-spun film with a uniformly dispersed conductive filler of graphene and CNTs in an elastomer [226]. Liu’s team reported a pressure sensor based on a nanofiber-reinforced graphene film [236]. The annealed electro-spun polyacrylonitrile (a-PAN) nanofibers with a conjugated structure were attached to the high-transparency thin graphene layer without using an adhesive through π–π interaction, ensuring high transparency of the film (≥ 94%, 600 nm).

**Fig. 11:**
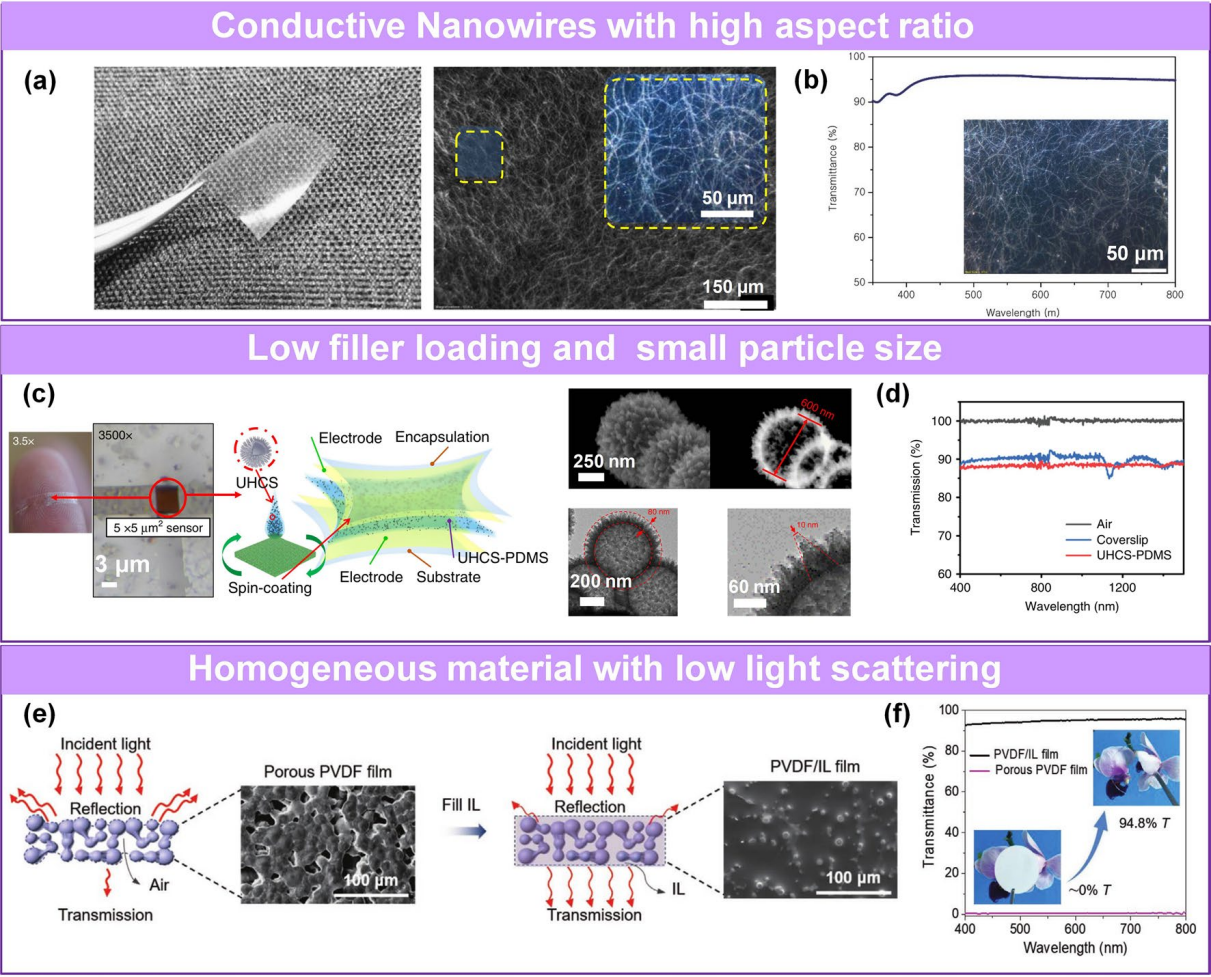
Pressure sensors with high transparency. **a** Transparent Ag NP electrode with high aspect ratio and **b** transmittance [93]. Copyright (2019) The Authors. **c** Photo, TEM image and **d** transmittance of transparent pressure sensors based on small size particles with low fill content [83]. Copyright (2020) The Authors. **e** Transparent transition mechanism of ionic liquids filling with porous film. **f** Comparison for the transmittance of ionic liquids before and after filling [235]. Copyright (2020) Wiley–VCH

Similarly, employing conductive materials with intrinsic high aspect ratio and high surface conductivity (e.g., metal nanowires) can decrease critical volume fraction of conductive layer to ensure a successive percolation network and maintain high transparency (Fig. 11a-b) [93]. Wu’s team reported a quantum effect-based transparent pressure sensor with less than 1.5 wt% urchin-like hollow carbon spheres (UHCSs) dispersed in PDMS (Fig. 11c-d) [83]. The high transparency was attributed to the low fill content and the small particle size of the UHCSs.

It is well known that a rough surface and inhomogeneous medium interface cause light scattering and reduce transparency. Liu et al. reported highly transparent iontronic pressure sensors created by filling porous PVDF film with ionic liquids (ILs) (Fig. 11e) [235]. Since the ILs had a refractive index (n = 1.41), which is approximately equal to that of the PVDF (n = 1.42), filling the pores with ILs produced a smooth surface and reduced light scattering, resulting in 94.8% transparency. This mechanism of obtaining a transparent sensor can be extended to other systems. A pressure sensor with electrodes consisting of ultra-thin transparent porous Ag NWs/polyimide (PI) film exhibited 90.4% transparency (Fig. 11f).

#### Pressure Sensors Capable of Directional and Selective Sensing

**Fig. 12:**
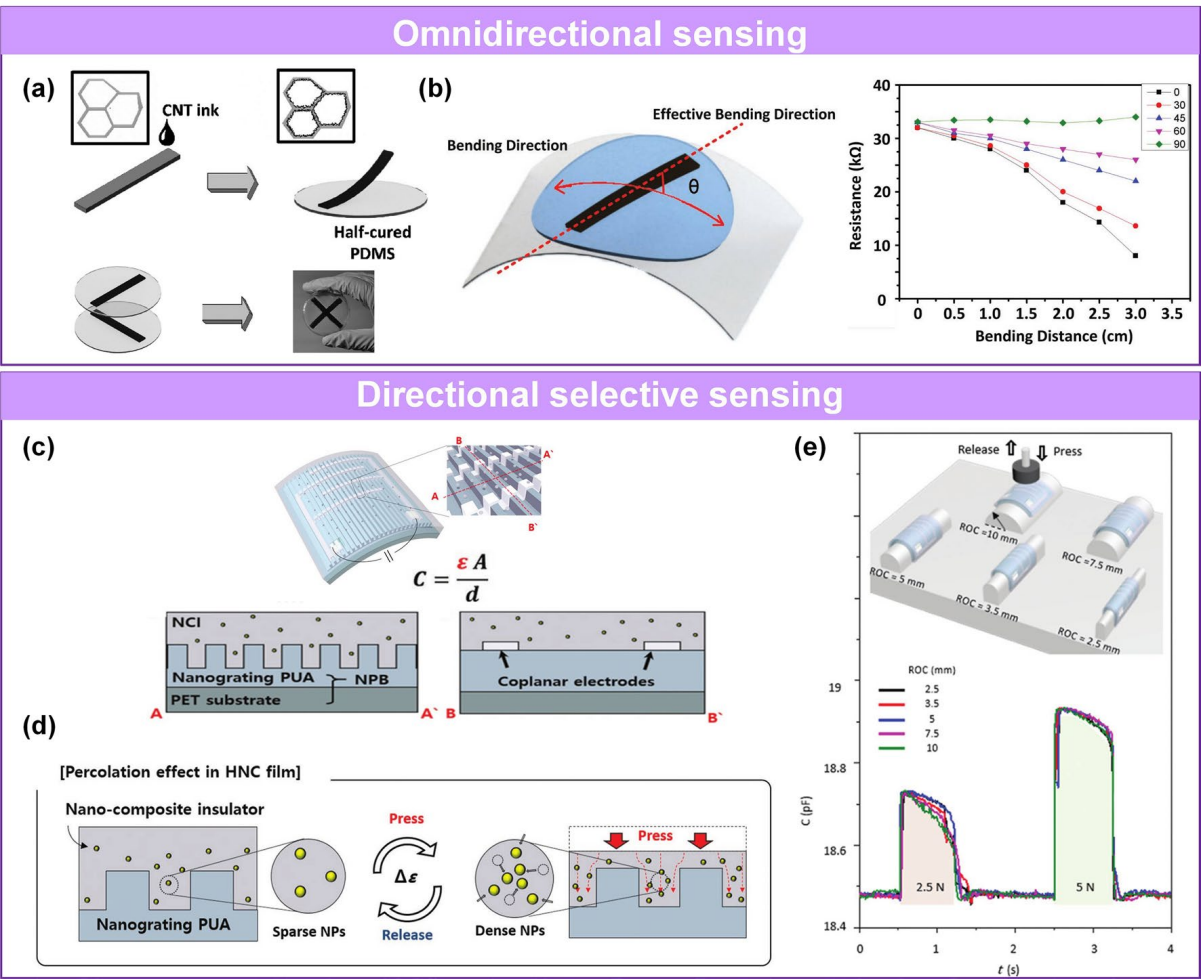
Pressure sensors toward directional-selective sensing. **a** Fabricated process and **b** working principle of two orthogonal CPSS-based bending sensor [70]. Copyright (2016) Wiley–VCH. **c** Schematic illustration of the bending-insensitive sensor based on coplanar electrodes. **d** Schematic diagram of dielectric material variation under pressure. **e** Bending test under certain pressure [227]. Copyright (2018) Wiley–VCH

Some pressure sensors are capable of selective/specific direction [227, 238], multi-dimensional [70, 239, 240] or omnidirectional [70, 239] signal detection through different structural designs. Ko’s team demonstrate a multidimensional mechanical sensor composed of two layers of prestrained silver nanowire percolation network with decoupled and polarized electrical response in principal and perpendicular directional. Two crossing strain sensors with anisotropic conductive film can independently detect the mechanical change of transverse and longitudinal axis [240]. Chen et al. fabricated a sensor capable of detecting omnidirectional bending and pressure by employing two orthogonal CNTs–PU sponge strips (CPSS) in the x-axis and y-axis directions (Fig. 12a-b) [70]. The bending distance and bending direction can be calculated using a coordinate system. The two functional layers respond to pressure and deformations with different levels of conductivity. Moreover, since only a pressure load produces a triboelectric response, the sensor can be used to distinguish between pressure and bending. FEA was used to determine the resistances of the functional layers. Omnidirectional sensing is suitable for detecting signals with multiple degrees of freedom in practical applications. Yoo et al. proposed a bending-insensitive capacitive pressure-touch sensor composed of a nanostructure plastic base, coplanar electrodes, and a dielectric polymer layer with Ag NPs [227]. The sensor is bending-insensitive sensing because the electrodes are located on the same plane near the neutral plane of the hierarchical nanocomposite (HNC) film (Fig. 12c-e).

## Microstructure Fabrication Strategies of Pressure Sensors

The method, scalability, and the stability and uniformity of the synthetic materials are important factors in the preparation of pressure sensors [129]. The preparation method depends on the application, material, equipment, and technical expertise. Commonly used methods to microstructured sensing layers include spray coating [150], dip-coating [28, 130, 138], vacuum filtration [241], and chemical vapor deposition [106, 242]. We summarize several examples of methods reported in the literature, focusing on self-assembly [26, 111, 114, 243, 244], patterning [115–117], and assisted synthesis [64, 66, 118, 119, 121, 134, 245, 246].

### Self-assembly Synthesis of Structured Sensing Materials

**Fig. 13:**
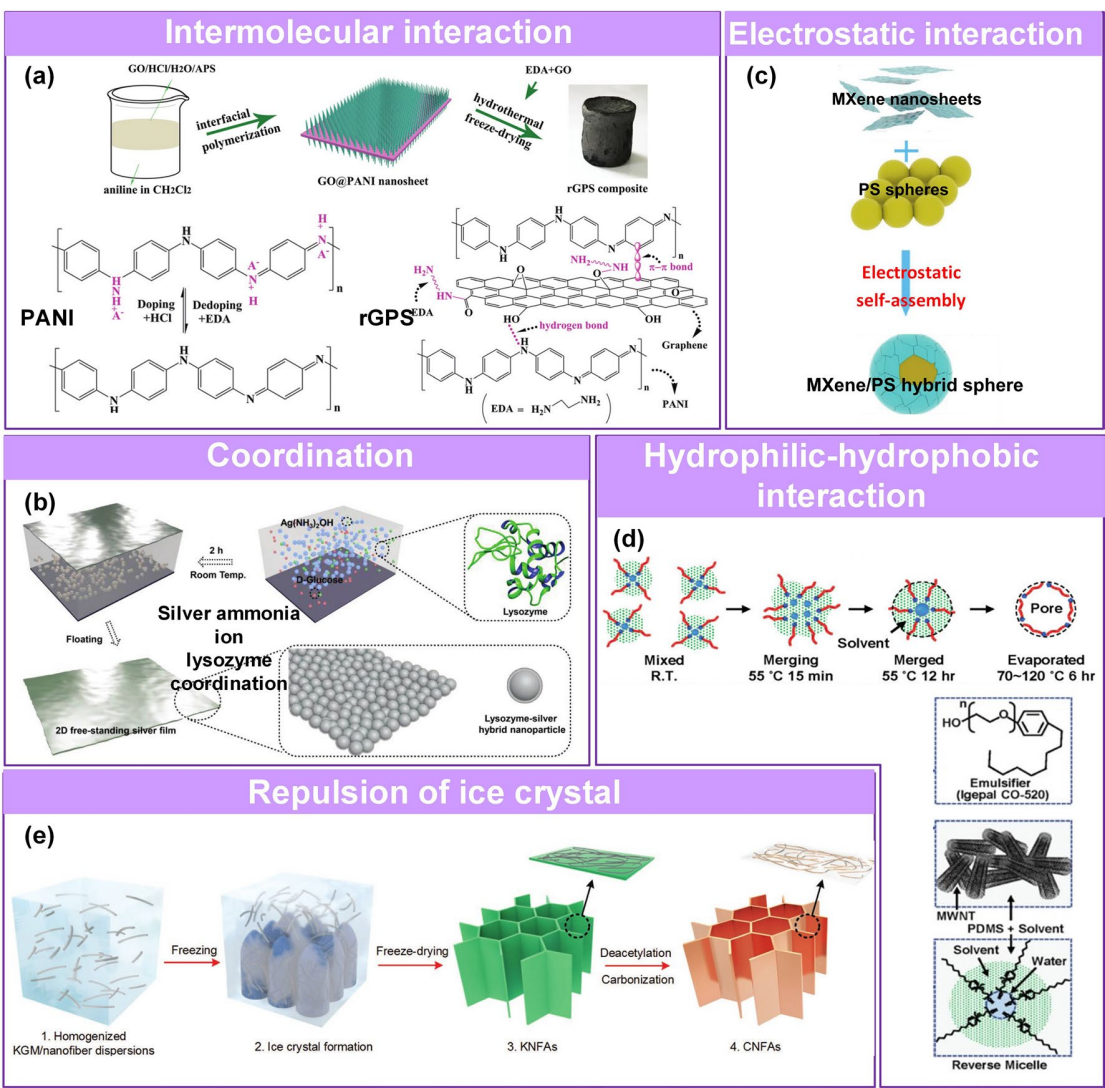
Self-assemble synthesis of structured sensing materials. Self-assembly process based on **a** Intermolecular interaction of the interactions of π–π interaction action and hydrogen bond between GO sheets and PANI nanowires [111]. Copyright (2019) Royal Society of Chemistry. **b** Coordination of Ag NPs and protein at the air/water interface [112]. Copyright (2018) Wiley–VCH. **c** Electrostatic interaction between PS sphere and MXene [113]. Copyright (2019) Wiley–VCH. **d** Hydrophilic-hydrophobic interaction to form micelles [26]. Copyright (2014) Wiley–VCH. **e** Honeycomb-shaped structure guided by growth and repulsion of ice [114]. Copyright (2016) Wiley–VCH

Self-assembly can be based on attractive or repulsive forces. It refers to a spontaneous process driven by intermolecular interaction (π–π interaction [111], hydrogen bonding [247], and van der Waals force [244]), electrostatic interaction [113, 248], coordination [112], hydrophilic–hydrophobic interaction [26, 249], ice crystal growth repulsion (ice-templated freezing) [114, 250], and other factors. Huang et al. reported a piezoresistive sensing sponge assembled with PANI and GO based on strong π–π interaction, hydrogen bonding, and electrostatic interactions [111]. The PANI was grown uniformly on the GO sheet, producing moderate wrinkles. Ethanediamine (EDA), a reducing agent of GO, was used to improve the GO’s conductivity. An ordered 3D network with a high mechanical strength was used as a crosslinking agent. The rGO/PANI sponge with a porous architecture was obtained after GO reduction and freeze-drying of the composites (Fig. 13a). Qin et al. developed metal–organic protein-bonding films based on coordination [112]. Silver ammonia ions were reduced by d-glucose to form silver nuclei, which were grown into Ag NPs via in situ accumulation (Fig. 13b). The obtained Ag film had a purity of 98% at the air/water interface with excellent flexibility and conductivity. The ultrathin protein-bonding layer functioned as a key mediator to tune the silver conductance dynamically in response to external pressures and strains.

Gao’s group fabricated hollow MXene sphere/rGO aerogel composites based on electrostatic adsorption for use as a pressure sensor [113]. The negatively charged MXene sheets were adsorbed on the positively charged polystyrene (PS) microspheres to form core–shell hybrid spheres based on electrostatic adsorption. The PS microspheres were removed via a heat treatment (Fig. 13c). The hollow structure increased the contact area under pressure. Additionally, some researchers have fabricated structured sensing materials based on hydrophilic/hydrophobic interactions. Jung et al. fabricated a porous pressure-sensitive rubber (PPSR) by a reverse micelle method [26]. The reverse micelles in the form of water droplets were surrounded by emulsifiers consisting of a mixture of PDMS/organic solvent/multiwalled CNTs. The components migrated, merged, and evaporated during the process, resulting in a porous sponge-like structure (Fig. 13d). The PPSR film and the conductive carbon fabric can be attached to create a large-sized pressure-sensitive fabric, making it suitable for use in pressure-sensitive clothing.

Ice-templated freezing, known as freeze-casting, is a self-assembly method consisting of ice crystal growth and the repelled active materials on the surface, forming a regulated lamellar or porous structure [114, 243, 250]. Aerogels can be prepared by freeze-drying due to the ordered growth of the ice crystals and its exclusion effect, where some ice nucleating agents can be further introduced to adjust the structure, size, and growth speed of ice crystals [251]. It is worth noting that aerogels used in pressure sensors must be able to recover without collapse. Si et al. developed ultralight carbonaceous nanofibrous aerogels (CNFAs) with a honeycomb structure by combining sustainable konjac glucomannan (KGM) extract and SiO_2_ nanofibers [114]. After freezing the KGM/nanofiber mixture, the KGM was repelled and accumulated at the edge of the growing ice crystals. The carbonaceous nanofibrous networks consisting of SiO_2_/carbon core–shell nanofibers were obtained after freeze-drying and heating. The fibers in the cell walls were tightly stacked in a honeycomb structure to improve strength and specific elasticity (Fig. 13e).

Microstructure fabrication strategies of microstructure based on intermolecular interactions, coordination, and electrostatic interaction are categorized as attractive self-assembly processes, whereas those based on hydrophilic–hydrophobic interactions and ice crystals are classified as repulsive self-assembly processes. Self-assembly produces structures with regular morphology.

### Patterned Synthesis of Structured Sensing Materials

**Fig. 14:**
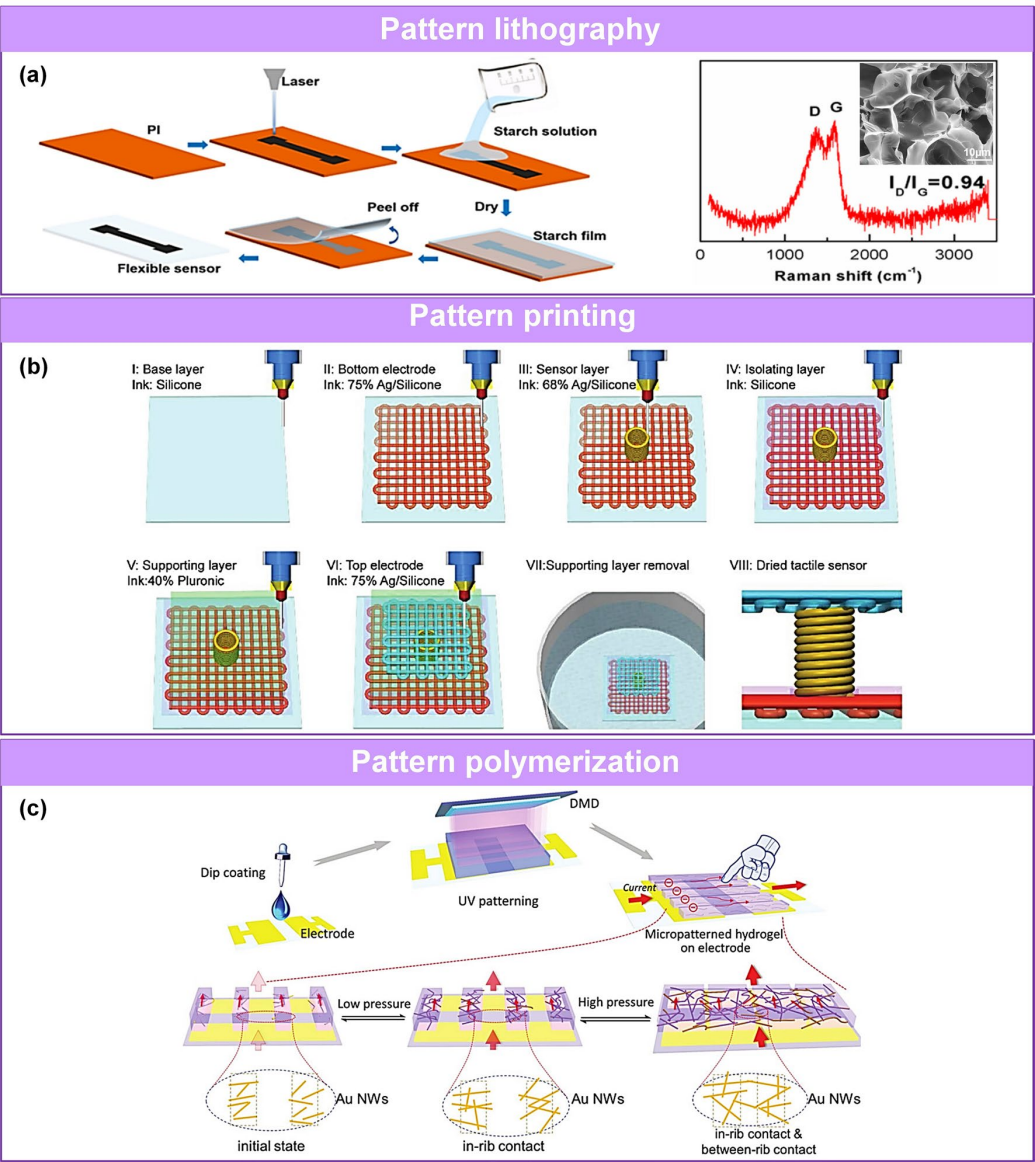
Patterned synthesis of structured sensing materials. **a** Construction process of sensor based on laser-treated PI film to starch film [115]. Copyright (2020) American Chemical Society. **b** The tactile sensors fabricated by pattern printing [116]. Copyright (2017) Wiley–VCH. **c** The patterning composite hydrogel based on photopolymerization of PAAm [117]. Copyright (2018) Wiley–VCH

Patterning methods represented by lithography [115, 132], printing [116, 252, 253], and polymerization [117] are the most commonly used strategies for preparing a patterned microstructure of active sensing materials used in pressure sensors. In the literature, the fabrication ease is often compared to that of lithography, which requires specialized equipment and is suitable for the assisted synthesis of microstructure sensing materials. Liu et al. proposed an e-skin based on laser-induced porous carbon on a starch film [115]. The PI film was carbonized by a laser treatment, which is a simple method to generate conducting networks with programmable patterns. Then, a starch film was used to support the carbonized layers (Fig. 14a). By adjusting the wavelength and power of the laser, carbonized porous structures with different apertures and thicknesses could be obtained.

Several printing methods have also been used to obtain a patterned design of active materials [116, 253, 254]. 3D printing is suitable for the construction of complex structures and custom products [116]. 3D printing uses a layering method to fabricate complex structures by layer-by-layer deposition without using templates [255–259]. This method is ideal for complex or curved surfaces. McAlpine’s group demonstrated the design and fabrication of stretchable tactile sensors composed of a base layer, a sensor layer, two electrode layers, an isolating layer, and a sacrificial supporting layer (Fig. 14b) [116]. They utilized nanocomposite ink optimization, 3D imaging, and multimaterial 3D printing. The unique composition of the sensor makes it possible for the detection of different human movements (finger pressing and bending) and the measurement of the radial pulse. The one-pot multimaterial 3D printing process enables the integration of various functional inks into the 3D sensor and provides a conformal design and high performance. For the 3D printing method, non-conductive polymer material is usually used for backbone structuring, and successive post processing is needed to add the conductive layer for their application in electronic devices [260]. Xia et al. reported a flexible piezoresistive sensor based on 3D-printing and spray-coating [260]. A hollow microcylinder structure flexible substrates is firstly fabricated by 3D-printing using photosensitive resin, and then, Au nanoparticles were spray-coated on the microstructured substrates to form the sensing layer. Sensors with the spraying time of 80 and 100 s show similar sensitivity, which can reach 419.622 kPa^−1^ in the pressure < 100 Pa. Kamat et al. constructed an elastomeric body-centered cubic (BCC) lattice structure through stereolithography (SLA) 3D-printing [261]. The lattice was dip-coated to deposit graphene nanoplatelets conductive layer and then assembled into a piezoresistive pressure sensor with good performance. The novel approach outlined in this work offers greater control over the microstructure and can be used to fabricate sensors with tunable properties.

Pattern polymerization is also popular for the construction of some patterned microstructures. Yin et al. reported a piezoresistive sensor based on UV polymerization-induced patterning of a micro-rib supramolecular hydrogel [117]. Au NWs were mixed with the polyacrylamide (PAAm) hydrogel to enhance conductivity. For the micropatterning of the sensing layer, the hydrogel was photopolymerized at the target position by patterned UV light to form microribs (Fig. 14c). The fabricated pressure sensors with a microrib structures exhibited both inner-rib and rib-to-rib contacts of Au NWs, enabling the tailoring of the sensitivity and operating range with different widths and spacing of microribs.

### Assisted Synthesis of Structured Sensing Materials

Sensing materials with unique structures can be fabricated using assisted synthesis methods, including a mechanical force-assisted [66, 109], electric field-assisted [118, 134], magnetic field-assisted [119], gas bubble-assisted [27, 64, 142], and template-assisted synthesis [121].

**Fig. 15:**
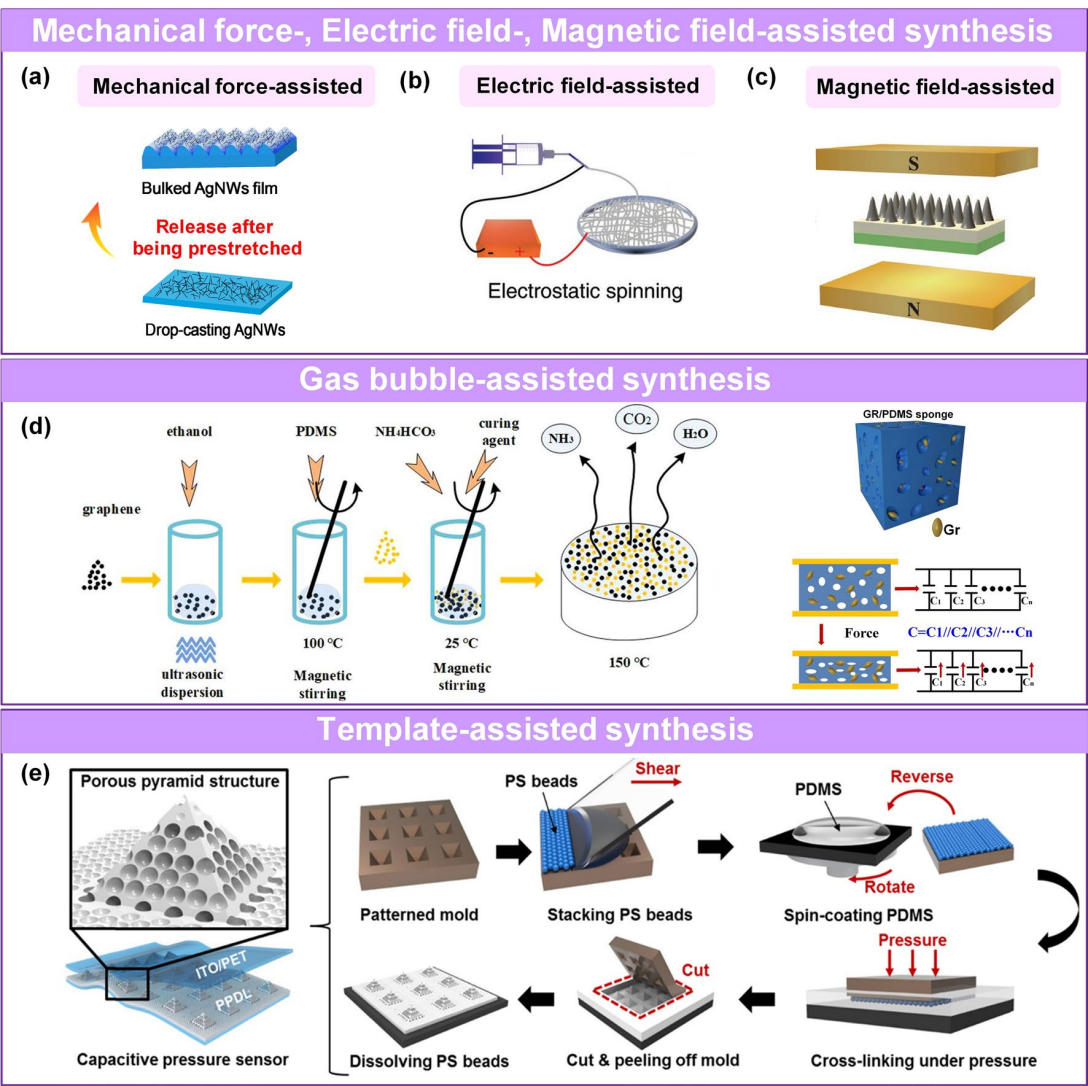
Assisted synthesis of structured sensing materials. **a** Fabrication process of wavelength wrinkles based on pre-stretched and release [66]. Copyright (2017) American Chemical Society. **b** Schematic illustration of the preparation of silk nanofiber membranes by electrospinning [118]. Copyright (2019) Wiley–VCH. **c** The microneedles formation of magnetic particles under magnitude [119]. Copyright (2020) Wiley–VCH. **d** Porous structure based on gas-assisted self-foaming [64]. Copyright (2019) The Authors. **e** Porous pyramid structure fabricated by pyramid structure of silicon mold and PS beads sacrificial template [121]. Copyright (2019) American Chemical Society

Some specific microstructures can be fabricated by mechanical force-assisted synthesis [109, 197]. Shuai et al. fabricated a capacitive pressure sensor with a buckled electrode and dielectric layer via a pre-stretching strategy (Fig. 15a) [66]. PVDF film and PDMS substrate coated with Ag NWs were used as a dielectric layer and top electrode, respectively. After a plasma treatment with dry low-pressure air, the surface of the pre-stretched PDMS film could be bent and was used to prepare microarray molds. The Ag NWs were transferred and embedded in the flexible microarray substrate to establish the elastomeric electrode. A capacitive pressure sensor with high sensitivity, fast response, low detection limit, and high flexibility was obtained by combining the upper electrode, dielectric layer, and bottom microarray electrode into a sandwich structure.

The electric field can also provide strong guidance for material fabrication, such as electrospun [118, 134] and electrochemical deposition [105]. Electrospinning is a strategy that uses an electric field to produce polymer filaments with different diameters by adjusting the nozzle shape, the distance between the capillary tube and the collection screen, the motion path of the collection device, and the potential and adding an auxiliary electrode. Typically, a 2D membrane or 3D fabric is obtained by electrospinning the fibers. Hou et al. fabricated a biodegradable silk fibroin-based flexible film sensor with > 90% transmittance using electrospinning (Fig. 15b) [118]. The electrical properties and optical transparency of the film could be regulated by adjusting the deposition density and effective thickness. Similarly, Asghar et al. proposed a piezocapacitive pressure sensor with a magnetically induced microneedle structure (Fig. 15c) [119]. The sensor was sensitive to pressure and field signals. In this system, the curable magnetorheological fluid particle (MP) self-assembled into an MP chain and continued to grow into microneedles by applying a vertical magnetic field. The spacing, density, and aspect ratio of the microneedles and the sensor’s Young’s modulus and sensitivity could be changed by adjusting the intensity of the magnetic field (B curing) and the MP concentration. Under optimized conditions, the sensor achieved excellent sensing performance with a detection limit of 1.9 Pa, a sensing range of 0–145 kPa, and cyclic stability of 9000 cycles.

A top-down gas bubble-assisted synthesis method has been widely used to obtain porous structures. The pore size [142], wall thickness [120], and density [142] of a monolithic bubble cluster can be adjusted by changing the concentration of the precursor solution and the dosage of the foaming agent. Kou et al. fabricated a piezocapacitive sensor using a dielectric layer consisting of a graphene/PDMS sponge with a 20% concentration of the NH_4_HCO_3_ foaming agent and a 2% concentration of graphene (Fig. 15d) [64]. The porous structure endowed the sensor with a low detection limit of 5 Pa, a rapid response time of 7 ms, and a wide detection range up to 500 kPa. Similarly, some chemical processes also produce additional gases to induce additional microstructure and enrich the morphology of active materials [262].

The template method is a molding strategy, including template-directed synthesis and sacrificial template. As mentioned above, in situ growth [25], impregnated (sponges [37], paper [28], and textile [130]), and coating [150] are the method of template-directed synthesis. In addition, scholars design various templates loading active materials to create micro-/nano-structures (micro-domes, pyramids, columnar, etc.) in sensing materials. Self-sacrificial template involves dissolving (sugar, salt, sponge) [108, 245, 263], evaporating (microfluidic droplets, frozen ice crystals, water droplets of breath figure method) [84, 114, 264, 265], and gasifying (polymer microspheres, sponge) [113, 246], whose sacrificial templates are created using sugar [263], salt microparticles [108], polymer microspheres [113], sponges [245], or microfluidic droplets [84] distributed in the active matrix. These templates are subsequently dissolved by a solvent or pyrolysis to form porous or hollow structures. Yang et al. constructed active materials with a secondary structure using a pyramid mold and PS microspheres as sacrificial templates (Fig. 15e) [121]. Notably, although the template method is relatively simple, the complex process and the potential for breakage during peeling is needed to be considered.

## Applications of Microstructure Pressure Sensors

Microengineering has been used to fabricate capacitive, resistive, piezoelectric, and triboelectric pressure sensors with very high sensitivities, very low detection limits, large working ranges, high transparency, and selective sensing. These high-performing sensors meet the emerging requirements of pressure sensors and have been used in exciting demonstrations, including wearable electronics in health care [1, 2, 31, 34, 39, 103, 266], intelligent devices for smart homes [16, 50, 72, 205], digitizing sport [21, 51, 52, 267], wireless monitoring in security [17, 23, 53], and ML-enabled intelligent sensor [54–60].

### Healthcare Applications

**Fig. 16:**
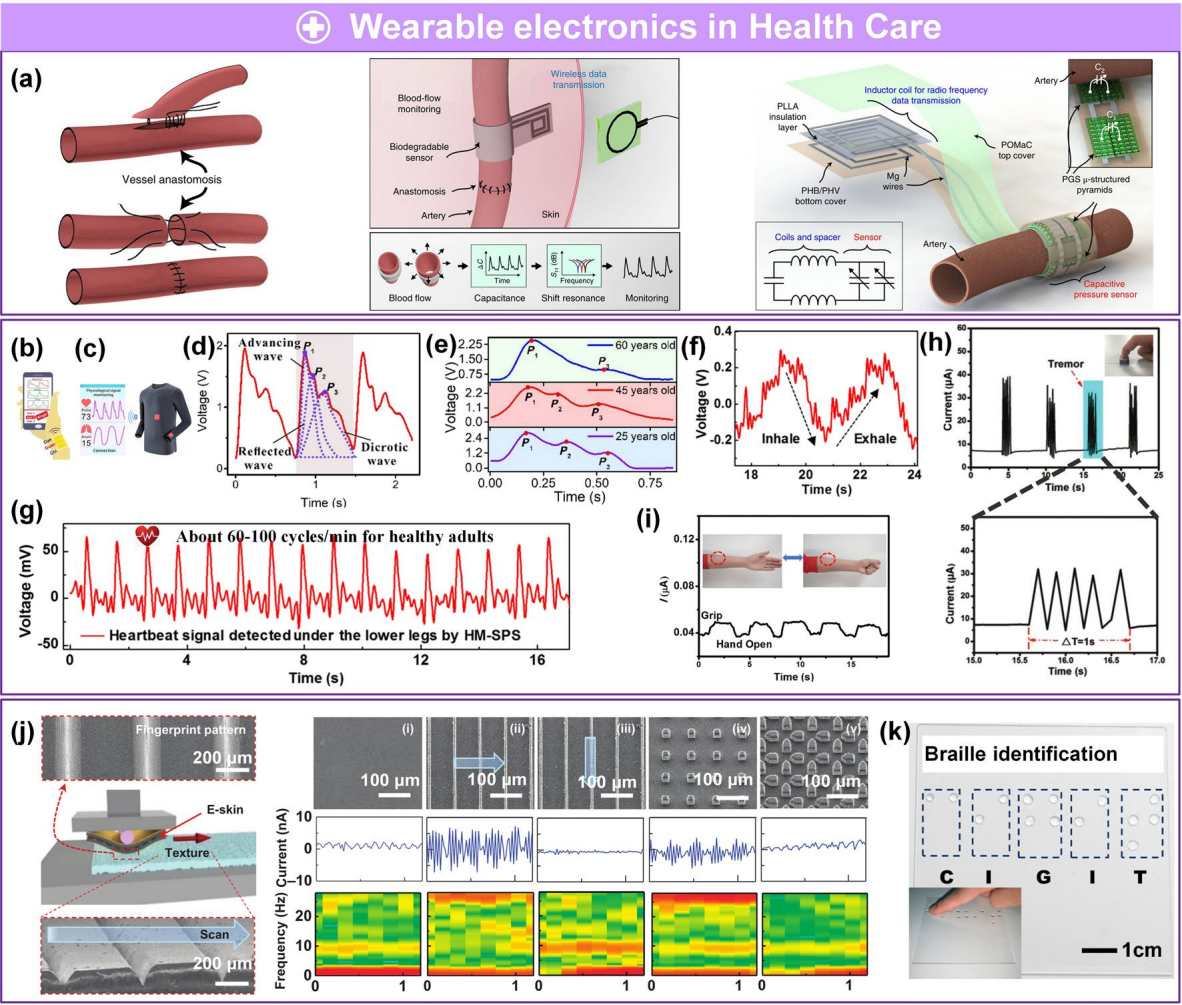
Applications as wearable electronics. **a** E-skin in vivo monitoring of blood flow [1]. Copyright (2019) Springer Nature. **b** E-skin in vitro [2]. Copyright (2018) Wiley–VCH. **c** Intelligent cloths [31]. Copyright (2020) The Authors. **d-e** Monitoring of physiological for beating of the pulse [31]. Copyright (2020) The Authors. **f** Monitoring of respiration and **g** heartbeat [32]. Copyright (2018) American Chemical Society. **h** Monitoring for tremor and **i** movement of human body [34]. Copyright (2019) American Chemical Society. Recognizing for **j** texture roughness [205] (Copyright (2015) The Authors) and **k** braille [103] (Copyright (2021) American Chemical Society)

As the population is aging and the demand for childcare increases, pressure sensors are increasingly used in health care to predict the health status of patients and prevent diseases by long-term monitoring of physiological signals, such as blood pressure [2], blood flow [1, 30], pulse beat [2, 6, 31, 32], heartbeat [32], respiration [31, 32], tremor [33–35], and body movement [36–38, 268] (Fig. 16). An e-skin can be used in vivo for effective and timely monitoring of changes in the blood vessel diameter and measuring blood flow during arterial pulsation after an operation to prevent disease recurrence and minimize postoperative discomfort (Fig. 16a) [1]. It is worth mentioning that the size of implanted electronics matches the tissues and organs, and the immune response, tissue growth, subsequent metabolism, and degradation are considered.

Additionally, pressure sensors are also widely used as e-skin attached to the body surface in the form of an electronic tattoo [269] and as smart clothing [26, 31], which has adequate ventilation, excellent washability, and comfort (Fig. 16b-c). These sensors can be used to monitor pulse wave signals to prevent cardiovascular diseases (e.g., the peak intensity decreases or no peak of the reflected wave (P_2_) occurs with increasing age, which is caused by the reflection of the waves due to a drop in the elasticity of the blood vessels [31]) (Fig. 16d-e). And these sensors can also be used for detecting respiratory and heartbeat signals to assist in diagnosing sudden death syndrome (Fig. 16f-g) [31, 32]. The output electrical signals change as the chest expands and contracts. The normal adult breathing rate is 12–20 times/min. Slow breathing (< 12–20 times min^−1^) is commonly associated with a reduced metabolic rate, shock, or an increase in intracranial pressure. In contrast, an excessive breathing rate (> 24 times min^−1^) is generally related to fever, heart failure, or bronchial asthma. Wearable sensors can also be used for monitoring tremors at a characterized frequency of 4–6 Hz [33, 34]) for early diagnosis of Parkinson's disease (PD) in real time and continuously (Fig. 16h) [35]. Wearable sensors are convenient for monitoring and correcting the sitting posture by attaching the sensor to the person’s back [39] and detecting movement signals of the human body (fingers, wrists, arms, knees, and other joint movements) to prevent accidental injuries (Fig. 16i) [36]. Notably, the sensor must be super-pliable and ultra-thin for in vivo and in vitro applications of e-skin to avoid restraining the skin and enable normal movements.

Interestingly, some sensors can also be used to distinguish the roughness of different substances [108, 205] and recognize braille [7, 103] based on different surface profiles (Fig. 16j-k). Some sensors with superior performance can detect pressures lower than that of a human finger [120], such as monitoring the evaporation of a drop of 40 uL ethanol [74] or detecting a mosquito landing [270] on a surface. Thus, these pressure sensors show significant potential for new applications.

### Smart Homes Applications

**Fig. 17:**
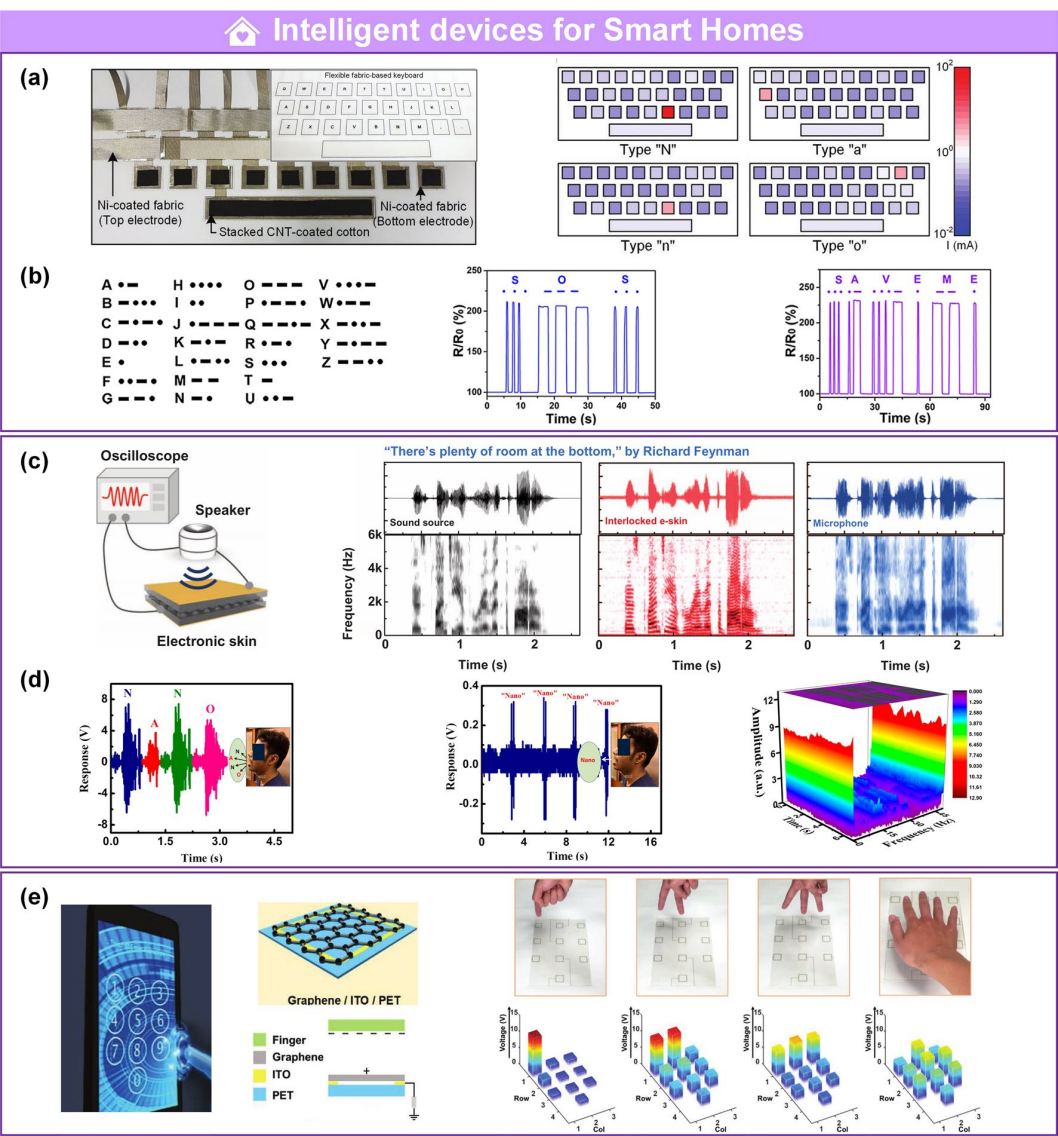
Application for smart homes. **a** Pressure sensor as touch control keyboard with different press level [50]. Copyright (2019) Wiley–VCH. **b** Pressure sensor as compile Morse code with variable pressing time [271]. Copyright (2021) American Chemical Society. **c** Pressure sensor as acoustic detector [205]. Copyright (2015) The Authors. **d** The case of “Nano” pronunciation measured by pressure sensor and respective short-time Fourier transform-processed 3D spectrograms [49]. Copyright (2020) American Chemical Society. **e** Pressure sensor as touch control screen [72]. Copyright (2019) Wiley–VCH

Pressure sensors are widely applied as intelligent devices in smart homes to facilitate various aspects of life [16]. These electronic devices typically operate based on the pressing intensity [50], duration and interval [44, 271]), acoustic control [49, 205], and mapping distribution [16, 72]. Pyo et al. fabricated a flexible, pressure-sensitive keyboard capable of matching letters by distinguishing the applied pressure ranging from a soft touch (ΔI/I_0_ < 10^3^) to a hard touch (ΔI/I_0_ > 10^3^) (Fig. 17a) [50]. Morse code detection was achieved based on the duration of the pressure (Fig. 17b) [34, 271]. Sound is a longitudinal wave [97], driving the air along the direction of propagation vibrates near the equilibrium position and leading to the formation of alternating waves. Voice control devices have great potential in smart homes based on considerable recognition (Fig. 17c-d) [205]. Some devices combined with pressure sensors can be operated with or without contact, and the results can be displayed as a spatial map. Tang et al. reported a triboelectric sensor integrated into a mobile phone screen, enabling contactless control and multipoint detection (Fig. 17e) [72].

### Digitizing Sports Applications

With the upsurge of modern competitive sports, an increasing number of athletes and coach teams carry out training according to scientific customized personality planning. The progress of artificial intelligence, big data and cloud computing provide new application opportunities in building a sports database. These sports data, collected by sensors, can provide feedback for athletes with real-time physiological state and sports effect [20].

**Fig. 18:**
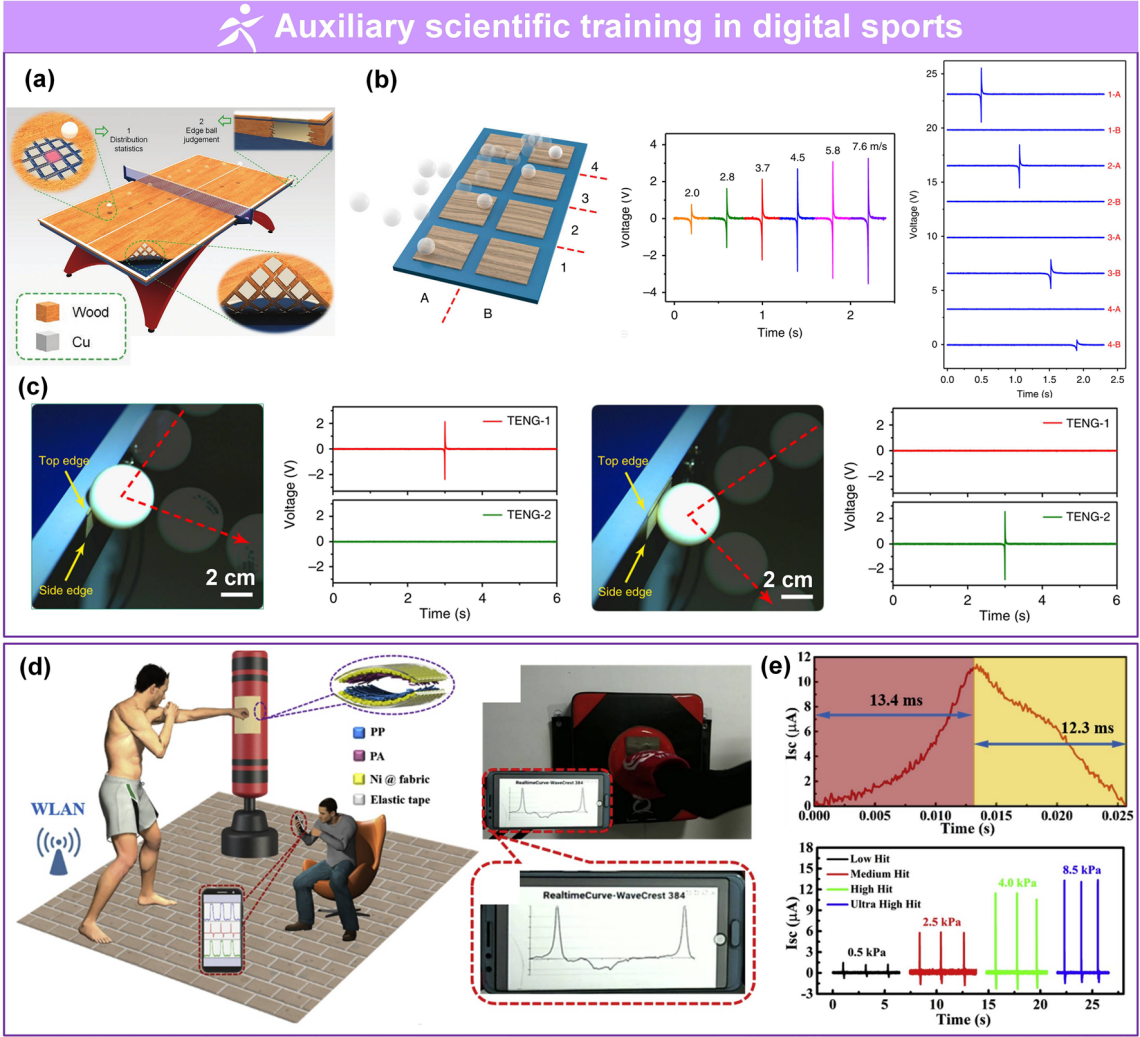
Application for digitizing sports. **a-c** Smart table tennis for point analysis [21]. Copyright (2019) The Authors. **d-e** Pugilism training monitor [51]. Copyright (2019) Elsevier

In the field of intelligent sports, in addition to the similar role of monitoring physiological signals in health care, pressure sensors can also be used for the training statistics. Luo et al. designed a wooden TENG array with a single electrode that can be mounted on a table to serve as an intelligent table tennis platform (Fig. 18a) [21]. The electrical signal of the ball touching the table can be used to 1) compile statistics and identify the ball’s impact velocity, drop location, drop sequence, and the probability of the drop point (Fig. 18b); 2) detect an edge ball using two single-electrode TENG on the top and side of the table (Fig. 18c). Additionally, as an alternative strategy for high-cost and bulky high-speed camera, Peng et al. fabricated a textile TENG attached to sandbags and pugilism targets for the monitoring of the strength and quantity of punch with a fast response (Fig. 18d-e) [51]. Similarly, some pressure sensors developed for intelligent sports in golf [267], baseball [52] and other projects also have the same digitizing sports applications [20].

### Wireless Monitoring in Security Applications

**Fig. 19:**
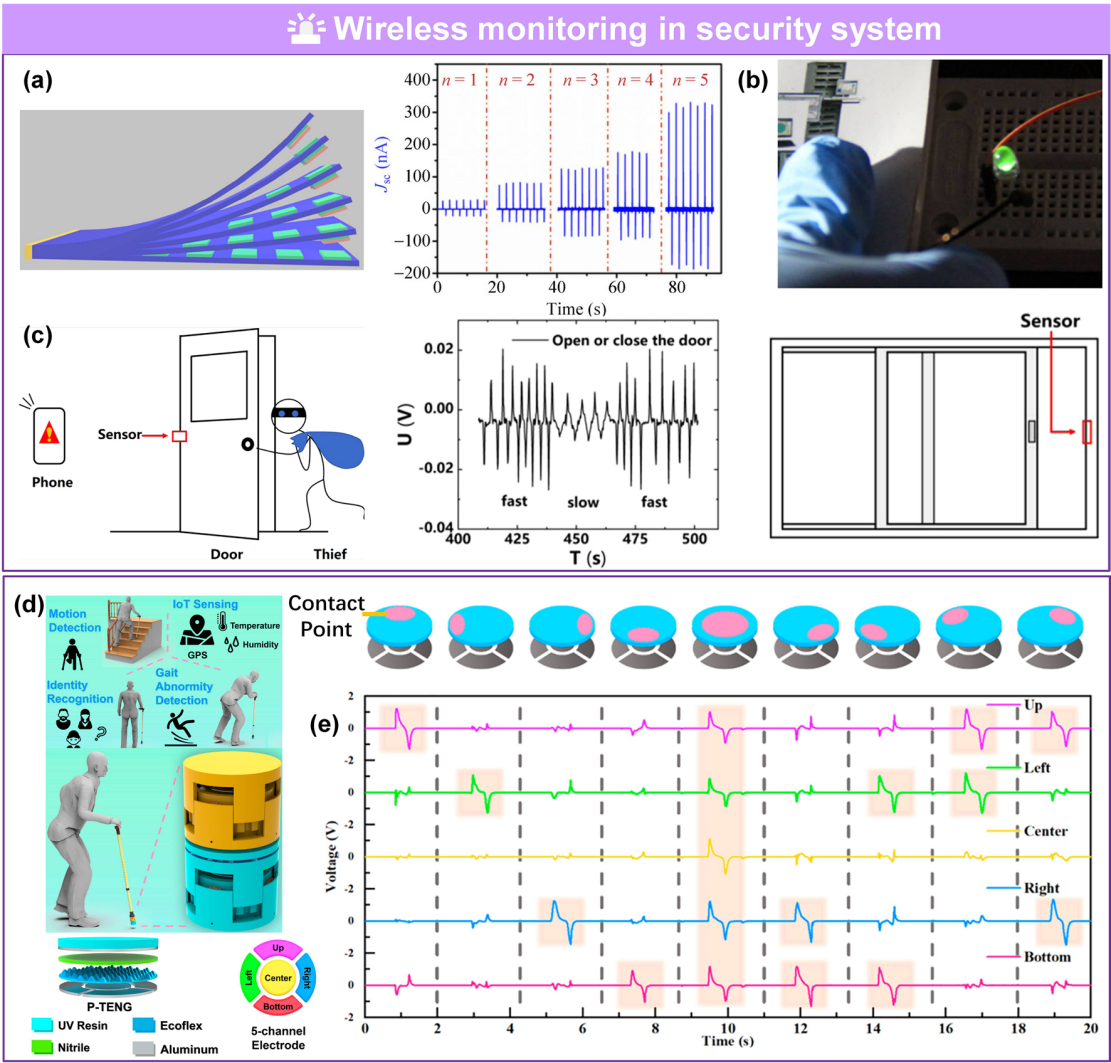
Application for wireless monitoring in security. **a-b** Page mark and theft-alarm system [23]. Copyright (2014) Springer Nature. **c** Anti-theft system for monitoring of door and window [53]. Copyright (2020) American Chemical Society. **d-e** Caregiving walking stick for locating and motor monitoring [17]. Copyright (2021) American Chemical Society

There is a growing trend for pressure sensors to be applicated in wireless monitoring of security. We divide the monitoring function into infrastructure safety [272, 273] and personal danger prediction. Wang and co-workers designed a document monitoring system in 2014 for real-time recording of browsing, page marking, and an anti-theft alarm system by employing a TENG operated in the vertical contact-separation mode and sliding mode (Fig. 19a-b) [23]. Different outputs were used to locate the opened page and record the page number. Liu et al. reported a piezoelectric single electrode sensor, which can be installed in the door, window or safe box, the opening of related positions can be monitored and can be used to warn the user to check whether there is theft or illegal intrusion (Fig. 19c) [53].

Pressure sensors show the potential of being an intelligent aid for a better life with safety and adequate autonomy especially for motion-impaired users. Lee and co-workers designed an intelligent walking stick with built-in GPS system for movement monitoring and assessment of motor disorders assessment [17]. The walking stick with five individual electrodes can detect the user’s gait characteristics based on the contact point, force, and sequence (Fig. 19d-e). The TENG that transforms the linear motion into a rotational motion produces a highly efficient output for ultralow frequency human motion.

### ML-Enabled Intelligent Sensing Platforms

ML is an interdisciplinary subject involving statistics, probability, approximation and algorithm theory [55, 56, 274]. ML includes the parts of collecting data, preparing data, selecting a model, training, evaluation, parameter adjustment, inference and prediction. The collected data are mainly divided into three parts: training set (used to train the model), verification set (to ensure that the model is not over fitted) and test set (to evaluate the effect of the model). The basic idea of ML is to abstract practical problems into mathematical problems and then solve it by dealing with mathematical problems. The development and maturity of ML are profoundly affecting the sensing technology [54–60, 275] and sensors with new function emerges, such as a deep-learned skin sensor for decoding the epicentral human motions [54, 60] and intelligent tactile textiles by learning human–environment interactions [57, 58]. They have promoted the mode of training and inference for accurately identify and sensing correction. The well-trained neural network models can create reasoning computational sensing systems and decode various behaviors through iteratively analyzing the data-driven sensing results.

**Fig. 20:**
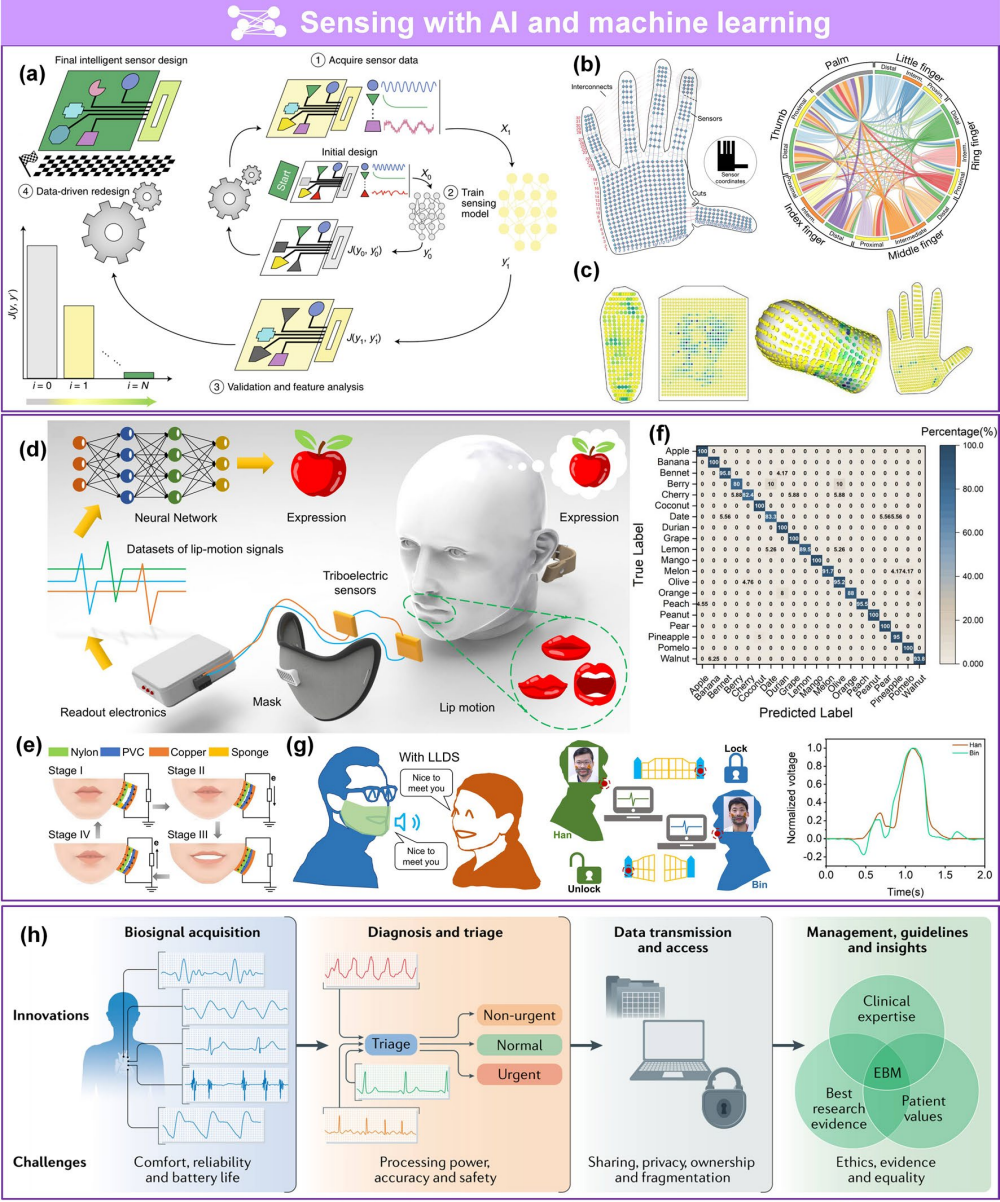
Application of ML-enabled intelligent sensing platforms. **a** Overview of ML-enabled intelligent sensor design [55]. Copyright (2021) Springer Nature. **b** The relative correspondences between different parts of the hand [57]. Copyright (2019) Springer Nature. **c** Learning human–environment interactions using conformal artificial robot skin, vest, sock and glove [58]. Copyright (2021) Springer Nature. **d-g** lip-language decoding system [59]. Copyright (2022) The Authors. **h** ML technology for scalable cardiovascular management [60]. Copyright (2021) Springer Nature

Ballard et al. discussed the computational sensing with a focus on intelligent sensor system design. They visaged a new generation of computational sensing system, which can reduce the data burden and improve the sensing ability (Fig. 20a) [55]. Through the iterative analysis of data-driven sensing results, low-cost and compact sensor implementation can be realized. Human beings can manipulate objects and tools with facility and accurately control the applied force. Extracting action features by learning from human–object interactions can aid the development of robots and prosthetics. Sundaram et al. researched the learning signatures of the human grasp with a tactile glove (Fig. 20b) [57]. The tactile glove and convolutional neural network (CNN) show that the uniformly distributed sensors on the hand can be used to identify individual objects, estimate their weight and explore the typical tactile patterns that emerge while grasping objects. Further, Matusik and co-workers designed conformal tactile textiles, in the forms of socks, vest, gloves and robot arm sleeve, to assist the calibration (Fig. 20c) [58]. The large-scale sensing textiles can record and analyze full-body and human–environment interactions. Lu et al. designed a decoding lip language system based on TENG with deep learning (Fig. 20d-e) [59]. The related confusion matrix can be used to match words with similar lip motion signals and guide the further improvement of TENG (Fig. 20f). Additionally, some interesting applications, such as help people with vocal cord lesions communicate effectively; identity recognition according to the characteristics of time domain and frequency domain for the host speaking, demonstrate its great feasibility and potential in new frontiers (Fig. 20g). Krittanawong et al. comprehensively reviewed the application of ML technology in accurate diagnosis, triage and management of cardiovascular diseases (Fig. 20h) [60]. Chandrabhatla et al. summarized the computational sensing technologies to categorize characteristic motor impairments of PD patients including tremor, gait, bradykinesia, and dyskinesia. Integration of AI or machine learning to the sensor provides new functionality that could not be possible for the traditional sensors [275].

As mentioned above, pressure sensors can realize many intelligent applications. In turn, artificial intelligence, especially ML, can also be used to guide the morphological engineering of the flexible pressure sensor or accelerate the sensor design [54–58]. ML has proven successful in predicting the relationships between material structures and device properties and can be an effective solution for screening a large inventory of materials that are potentially suitable for a specific application [55]. The built model can provide insights into the key material characteristics that are linked to the target properties of a sensor, which would enable a rapid computational screening of data bases to identify candidate material that are likely to possess the desired properties. Yang et al. developed a prediction model to realize automating the design of mechanical sensors [56]. A three-stage framework containing a support vector machine (SVM) classifier training, active learning loops and data augmentation, was to realize the define of the model’s boundaries, enrich the multidimensional dataset and cultivate the model’s prediction accuracy, respectively. Two-way tasks of automatic strain sensor design can be achieved by this machine learning-enabled prediction model, including high-accuracy sensor performance prediction based on a proposed fabrication recipe and recommendation of feasible fabrication recipes to obtain adequate strain sensors for monitoring a specific soft machine [56]. The final model-suggested strain sensors can be integrated into/onto various soft machines to realize real-time sensing capabilities.

## Conclusions and Prospects

In the past few years, tremendous progress has been made on flexible and wearable pressure sensors by the development of various novel materials/structures. In this review, we summarize the current development of morphological engineering technologies for flexible pressure sensors. Some typical and representative microarchitectures, including compressible interlayer structure [94–96], microrough structures [93, 102, 103], porous hierarchical structures [32, 34, 37, 61, 104–106], and multiscale hierarchical structures [25, 50, 63, 74, 82, 107–109], are comprehensively illustrated. These morphological engineering toward the related device performance, including high sensitivity, broad working range, stable sensing, low hysteresis, high transparency, directional-selective sensing, are prominent stated. A detailed analysis of the engineered microstructure materials with unique sensing performances and their fabrication techniques are compared. In addition, important applications in the areas of healthcare [2, 31, 39], smart homes [49, 50], digitizing sports [20, 21, 51], wireless monitoring in security [17, 23, 53], ML-enabled intelligent sensing platforms [54–60] have been discussed.

Future research work and efforts toward practical application and commercialization of these high-performance pressure sensors should focus on addressing the following challenges. (1) Pressure sensors with high sensitivity, a wide working range, and long-term durability are required in many practical applications. (2) For materials/devices, device standardization, modeling/simulation intelligent learning, and data-driven approaches to guide design [55, 56] are needed to understand the relationship between pressure sensor performance and microstructure (such as pore size, size distribution). (3) For real-time human health monitoring, as well as the applications in modern soft robots and artificial intelligence systems, a great number of multidisciplinary studies are needed to develop wearable sensing systems integrated with multi-functional sensors (such as temperature, pressure, physiological signals) [205] and with some additional performance (electromagnetic shielding [276] and self-healing [89, 277], sensing and actuating function [41, 237]), power systems (e.g., batteries) [263, 278], and real-time data transmission modules. (4) Some special artificial intelligence applications need high-density and low-cost pressure sensor arrays to effectively collect the signals of various parts, but there is still a lack of low-cost, reliable, and high-precision preparation methods to realize the high-pixel and large-area preparation of pressure sensors. The work in these areas will be an important step in bringing flexible and wearable pressure sensors to future practical applications.
